# The anti-tumour activity of DNA methylation inhibitor 5-aza-2′-deoxycytidine is enhanced by the common analgesic paracetamol through induction of oxidative stress

**DOI:** 10.1016/j.canlet.2020.12.029

**Published:** 2021-03-31

**Authors:** Hannah J. Gleneadie, Amy H. Baker, Nikolaos Batis, Jennifer Bryant, Yao Jiang, Samuel J.H. Clokie, Hisham Mehanna, Paloma Garcia, Deena M.A. Gendoo, Sally Roberts, Megan Burley, Alfredo A. Molinolo, J. Silvio Gutkind, Ben A. Scheven, Paul R. Cooper, Joanna L. Parish, Farhat L. Khanim, Malgorzata Wiench

**Affiliations:** a School of Dentistry, Institute of Clinical Sciences, College of Medical and Dental Sciences, The University of Birmingham, Birmingham, B5 7EG, UK; b Institute of Head and Neck Studies and Education (InHANSE), The University of Birmingham, Birmingham, B15 2TT, UK; c Institute of Clinical Sciences, The University of Birmingham, Birmingham, B15 2TT, UK; d West Midlands Regional Genetics Laboratory, Birmingham Women's and Children's Hospital, Birmingham, B15 2TG, UK; e Institute of Cancer and Genomic Sciences, College of Medical and Dental Sciences, The University of Birmingham, Birmingham, B15 2TT, UK; f Centre for Computational Biology, Institute of Cancer and Genomic Sciences, The University of Birmingham, Birmingham, B15 2TT, UK; g Moores Cancer Center and Department of Pathology, University of California San Diego, La Jolla, CA, 92093, USA; h Department of Pharmacology and Moores Cancer Center, University of California San Diego, La Jolla, CA, 92093, USA; i Present Address: MRC London Institute of Medical Sciences, Imperial College London, London, W12 0NN, UK; j Present Address: Sir John Walsh Research Institute, University of Otago, Dunedin, New Zealand

**Keywords:** Decitabine, Acetaminophen, Head and neck squamous cell carcinoma, Acute myeloid leukaemia, Epigenetic therapies, AA, arachidonic acid, AML, acute myeloid leukaemia, CI, combination index, CMAP, Connectivity Map, COX-2, cyclooxygenase 2, DAC, 5-aza-2′-deoxycytidine, DRI, dose reduction index, ERV, endogenous retrovirus, GSH, glutathione, HNSCC, head and neck squamous cell carcinoma, LOX, lipoxygenase, NAC, N-acetyl-cysteine, NAPQI, N-acetyl p-benzquinone-imine, PGE_2_, prostaglandin E_2_, PTGS2, prostaglandin-endoperoxidase synthase 2, ROS, reactive oxygen species, TXN, thioredoxin, TXNRD, thioredoxin reductase

## Abstract

The DNA demethylating agent 5-aza-2′-deoxycytidine (DAC, decitabine) has anti-cancer therapeutic potential, but its clinical efficacy is hindered by DNA damage-related side effects and its use in solid tumours is debated. Here we describe how paracetamol augments the effects of DAC on cancer cell proliferation and differentiation, without enhancing DNA damage. Firstly, DAC specifically upregulates cyclooxygenase-2-prostaglandin E_2_ pathway, inadvertently providing cancer cells with survival potential, while the addition of paracetamol offsets this effect. Secondly, in the presence of paracetamol, DAC treatment leads to glutathione depletion and finally to accumulation of ROS and/or mitochondrial superoxide, both of which have the potential to restrict tumour growth. The benefits of combined treatment are demonstrated here in head and neck squamous cell carcinoma (HNSCC) and acute myeloid leukaemia cell lines, further corroborated in a HNSCC xenograft mouse model and through mining of publicly available DAC and paracetamol responses. The sensitizing effect of paracetamol supplementation is specific to DAC but not its analogue 5-azacitidine. In summary, the addition of paracetamol could allow for DAC dose reduction, widening its clinical usability and providing a strong rationale for consideration in cancer therapy.

## Highlights

• Paracetamol works in synergy with DAC (decitabine) to reduce cancer cell viability.
• The synergistic effect is specific for decitabine and not observed for 5-azacitidine.
• Paracetamol allows for DAC dose reduction without inducing DNA damage.
• Paracetamol counteracts DAC-triggered activation of COX-2-PGE_2_ pathway.
• In the presence of paracetamol DAC induces mimicry of paracetamol overdose leading to oxidative stress.

## 1 Introduction

Aberrant DNA methylation patterns are common in most cancers, arise early in tumour development and are potentially reversible by hypomethylating agents [1]. 5-aza-2′-deoxycytidine (Decitabine or DAC) is a nucleoside analogue that incorporates into replicating DNA in place of cytosine where it traps and promotes the degradation of DNA methyltransferases (DNMTs) [2]. This results in two anti-cancer activities: methyl marks cannot be copied during DNA replication causing widespread DNA demethylation; and adducts are formed in the DNA leading to DNA damage and apoptosis [2]. DNA demethylating drugs are thought to de-repress epigenetically silenced tumour suppressor genes as well as demethylate endogenous retroviruses (ERVs), triggering an antiviral immune response and cancer cell death [[2], [3], [4]]. DAC has been approved by the European Medicines Agency (EMA) for treatment of acute myeloid leukaemia (AML) [5,6], while pre-clinical studies suggest it might also be effective in solid tumours [7]. However, the outcomes of clinical trials are highly variable, likely attributed to small sample sizes, lack of patient stratification, and inappropriate dosing and schedual [8].

Head and neck squamous cell carcinoma (HNSCC) originates from stratified squamous epithelium of the oral cavity and pharynx where the cells in the basal cell layer proliferate and replenish the suprabasal layers undergoing terminal differentiation [9]. HNSCC has a 5-year survival rate of ≤40%, highlighting a pressing need for new therapies [10]. Despite DNA methylation aberrations being common [11], the clinical evaluation of DAC potential in HNSCC is limited [12].

In solid tumours, DAC alone may not be curative, but favourable effects were observed when combined with other chemo- and immune-therapies [8]. However, it is not known whether the response to DAC could be enhanced by compounds not traditionally used in cancer treatment. In the current study, a custom-built library of 100 commonly used, cost-effective, off-patent drugs [13] was investigated for their ability to sensitize HNSCC cells to DAC treatment. Of the drugs tested, paracetamol was identified to work in synergy with DAC.

Paracetamol (acetaminophen) is the most commonly used analgesic and antipyretic in both Europe and the United States [14]. Paracetamol affects the cyclooxygenase (COX) pathway wherein arachidonic acid (AA) is metabolized to prostaglandin H_2_ (PGH_2_) by either constitutively expressed COX-1 (PTGS1) or inducible COX-2 (PTGS2) [15]. PGH_2_ is then converted, by respective prostaglandin synthases, into effector prostanoids (prostaglandins PGE_2_, PGF_2__,_ PGI_2_ and PGD_2_ or thromboxane TXA) which work through metabolite-specific G-protein coupled receptors to activate downstream pathways [15].

The COX-2-PGE_2_ axis is associated with inflammation, growth and survival and is thought to contribute to the ‘inflammogenesis of cancer’ [16]. Increased expression of *PTGS2* and production of PGE_2_ are found in many solid tumours, including HNSCC, and correlate with tumour stage, metastasis and worse clinical outcome, whilst low levels are associated with better response to chemotherapy [16,17]. Hence, COX-2 inhibitors have been tested for their anti-cancer therapeutic activity activity and showed potential as preventative agents [17,18]. In established tumours however, only overdose concentrations of paracetamol have demonstrated therapeutic potential [[19], [20], [21]].

Here we show that paracetamol can be used at clinically relevant concentrations to sensitize cancer cells (both HNSCC and AML) to DAC treatment, allowing for DAC dose-reduction.

## 2 Materials and methods

### 2.1 Cell lines and culture conditions

Five human HNSCC cell lines were used: SCC040 (German Culture Collection, DSMZ (#ACC660)), FaDu (ATCC (HTB-43)), VU40T (Prof H. Joenje (VU University Medical Centre, Amsterdam)), HN12 (Dr J.F. Ensley (Wayne State University, Detroit, MI)) and UDSCC2 (Dr Henning Bier (University of Duesseldorf, Germany)). The cell lines were authenticated using STR profiling (NorthGene, UK). HNSCC cell lines were maintained in DMEM (Sigma- Aldrich) supplemented with 10% fetal bovine serum (FBS) (Sigma-Aldrich), 1% penicillin-streptomycin (Gibco), 4 mM l-glutamine (Sigma-Aldrich), 1X non-essential amino acids (Life Technologies) and 1 mM sodium pyruvate (Life Technologies). Primary human oral keratinocyte (HOK) cells were purchased from Caltag Medsystems and cultured over Poly-l-Lysine in Oral Keratinocyte Medium supplemented with 1% oral keratinocyte growth supplement (all from ScienceCell) and 1% penicillin-streptomycin. AML cell lines (SKM-1 (from Dr Stefan Heinrichs, University of Essen, Germany) and HL-60 (ATCC, CCL-240)) were cultured in RPMI1640 medium (ThermoFisher Scientific), supplemented with 15% FBS (Sigma-Aldrich), 1% l-glutamine and 1% penicillin-streptomycin. All cell lines were regularly tested for mycoplasma using MycoAlert (Lonza).

### 2.2 Drugs treatments

All drugs were purchased from Sigma-Aldrich. 5-Aza-2′-deoxycytidine (DAC) was dissolved in either 50% acetic acid or in ≥99.9% dimethyl sulfoxide (DMSO); all other drugs, including azacitidine, were dissolved in DMSO. The treatments were carried out for 96h (HNSCC cells) or 72h (AML cells) using the relevant vehicle as a control.

### 2.3 Viability assay

Relative viability was determined in 96 well plates using the CellTiter-Blue Cell Viability Assay (Promega), including a minimum of triplicate wells per each sample and controls: vehicle, high concentration vehicle and ‘media only. Samples were normalized to a vehicle-only control. Sigma plot software (Systat Software Inc.) was used to generate sigmoidal, 4-parameter dose response curves after drug titrations. IC_50_ values were calculated using MyCurveFit (MyAssays Ltd.).

### 2.4 DAC sensitizing assay

A panel of 100 drugs (Drug Library FMC1) were administered at the reported peak serum concentrations (C_max_) [13]. Cells were treated with each drug alone or in combination with 500 nM DAC. The assay was performed blind with controls hidden within the panel and viability assessed as above.

### 2.5 Determining synergy

The Chou-Talalay method was employed to determine synergy [22] using constant-ratio matched titrations (0.125X, 0.25X, 0.5X, 1X, 2X, 3X, 4X, and 8X) of multiples of the C_max_ (500 nM for DAC, 132 μM for paracetamol, 3 μM for azacitidine) and assessed by cell viability. The CompuSyn software [22] was used to calculate combination index (CI) and dose reduction index (DRI) values.

### 2.6 Long treatment of AML cells

SKM-1 and HL-60 cells were subjected to four cycles of 72h treatments followed by 21 days withdrawal period (details in Supplementary Methods), the cells were counted at each passage using trypan blue (Sigma-Aldrich) staining and growth rates were calculated.

### 2.7 DNA dot blotting

DNA was extracted using the DNeasy Blood and Tissue Kit (Qiagen) and DNA dot blotting performed as described in Ref. [23] using a titration of DNA; detailed protocol and antibodies are provided in Supplementary Methods. Blots were normalized to methylene blue (Sigma-Aldrich) staining.

### 2.8 Western Blot analysis

Western Blot analysis was performed as described previously [13], with some alterations; protocol details and antibodies are provided in Supplementary Methods.

### 2.9 Giemsa-Jenner staining

VU40T cells were grown on coverslips for 96h, fixed in methanol and stained with Giemsa and Jenner (VWR) as previously described [13]. SKM-1 cells were transferred to a glass slide with a Cytospin 3 (Thermo Shandon) before fixation. Microscope images were taken using EVOS XL Core Imaging System or BX-50 Olympus with a 100x oil immersion lens.

### 2.10 Immunofluorescence analysis

Immunofluorescence analysis was undertaken as described in Ref. [4] with details in Supplementary Methods.

### 2.11 Apoptosis assay and cell cycle analysis

Apoptosis and necrosis were assessed using the Annexin V Apoptosis Detection APC kit (eBioscience, ThermoFisher Scientific); details in Supplementary Methods. For cell cycle analysis, cells were fixed in 70% ice cold ethanol and resuspended in 200 μl 50 μg/ml propidium iodide solution (Sigma-Aldrich) and 50 μl of 100 μg/ml RNase solution (Roche). The solutions were analysed by flow cytometry on a Cyan B FACS analyser (Beckman Coulter).

### 2.12 ELISA

The levels of PGE_2_, Leukotriene B_4_ (LTB_4_) and Cysteinyl leukotrienes (LTC_4_, LTD_4_, and LTE_4_) were assessed in cell media using respective ELISA kits (Abcam). The results were normalized to the corresponding CellTiter-Blue viability results or cell count.

### 2.13 Determining glutathione concentration

The glutathione levels were measured using the GSH-Glo Glutathione Assay (Promega) with 1 mM paracetamol used as a positive control. The results were normalized against the corresponding CellTiter-Blue cell viability or cell density.

### 2.14 NAC rescue assay

N-acely-l-cysteine (NAC, Sigma-Aldrich) was dissolved in water and neutralised using 1 M sodium hydroxide (Sigma-Aldrich). VU40T cells were treated with 2.5 mM NAC or an equivalent volume of vehicle for 48h. Following this, wells were washed with fresh media and cells were treated with DAC, paracetamol or both for 96h and cell viability was determined.

### 2.15 Assessment of reactive oxygen species (ROS) and mitochondrial superoxide (mitosox)

MitoSOX Red (Cat# M36008, ThermoFisher Scientific) was used to assess mitosox, while ROS were measured using carboxy-H_2_DCFDA (5-(and-6)-carboxy-2′,7′-dichlorodihydrofluorescein diacetate, Cat# C369, Invitrogen); details in Supplementary Methods.

### 2.16 Real-time quantitative PCR (qRT-PCR)

Total RNA was extracted using RNeasy Mini Kit (Qiagen), including DNase I digestion step (RNase-Free DNase set, Qiagen). 1 μg of RNA was reverse transcribed (iScript cDNA Synthesis Kit (BioRad)) and the cDNA was purified with QiaQuick PCR Purification Kit (Qiagen). Purified cDNA was quantified using Qubit dsDNA High Sensitivity Assay kit (Thermo Fisher Scientific) to support normalization. qRT-PCRs were performed on a LightCycler 480 II using LightCycler 480 SYBR Green (Roche). Additional information and primers’ sequences are provided in Supplementary Methods and Supplementary Table S1.

### 2.17 RNA sequencing

For each sample, three biological RNA replicates were pooled to make a library using the TruSeq Stranded mRNA Library Prep kit and sequenced using a NextSeq 500 (Illumina) in paired-end mode at 2x75 bases in the Genomics Birmingham facility (Birmingham, UK). Reads were aligned to the genome (hg19) using HiSAT2 and processed with bedtools to generate normalized coverage plots. Details on quantification and data processing are in Supplementary Methods.

### 2.18 Mouse xenograft study

The mouse xenograft study was performed as described previously [24] and details of the protocol are in Supplementary Methods. To examine the toxicity and anti-tumour efficacy of DAC and paracetamol, we utilized male NOD/SCID/gamma (NSG) mice (Charles River) in accordance with the UK Home Office Animal (Scientific Procedures) Act 1986 and approved by the local University of Birmingham Ethical Review Committee. 24 males were implanted with 5 × 10^6^ FaDu HNSCC cells suspended in serum-free medium and injected subcutaneously into the right flank. After three days the tumours established and mice were randomly allocated into four treatment groups on a 5 day on, 2 day off regimen: 0.4 mg/kg DAC in PBS via IP injection (or 0.2 mg/kg in the week three); 100 mg/kg paracetamol in PBS via oral gavage; DAC plus paracetamol as above; control (PBS) given both through the oral gavage and IP injection. Animals were monitored daily for signs of ill health and the tumours were measured. Upon culling, the tumours were excised and snap frozen for RNA extraction and subsequent analysis.

### 2.19 Quantification and statistical analysis

Statistical analysis and graphs were performed using GraphPad PRISM 8 software, unless stated otherwise. Details of the statistical tests used for each experiment are given in the corresponding figure legends.

### 2.20 TCGA data

Genetic alterations, gene expression and patient survival data were retrieved from The Cancer Genome Atlas (TCGA) via cBioPortal for Cancer Genomics [25]; the details are given in Supplementary Methods.

### 2.21 Drug perturbation signatures

Drug perturbation signatures were downloaded for the BROAD Connectivity Map dataset (CMAP) using the PharmacoGx package (version 1.14.0) [26] in R (details in Supplementary Methods).

### 2.22 Drug Set Enrichment Analysis

The effect of Decitabine, paracetamol, valdecoxib and azacitidine drug combinations on KEGG pathways and Gene Ontology Biological Processes was conducted using the Drug Set Enrichment Analysis (DSEA) server [27]. Significance of the perturbed pathways of interest was identified using the corresponding p-values per geneset; log_10_ (p-values) were plotted as heatmaps.

### 2.23 Data availability

RNA-Seq data: The data are deposited at the GEO repository, accession number GSE110045 and SRA, accession number SRP132039.

## 3 Results

### 3.1 HNSCC cell lines show bimodal sensitivity to DAC treatment

**Fig. 1:**
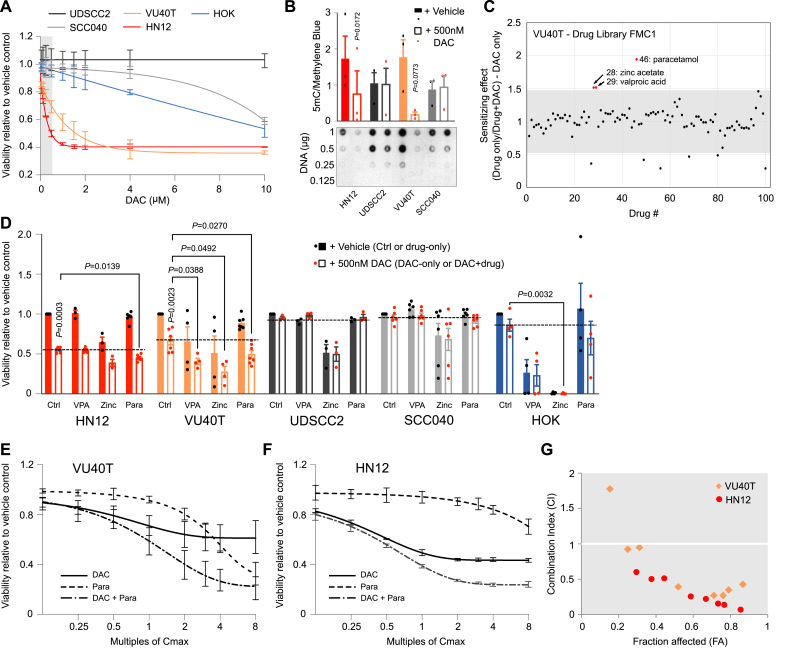
DAC therapeutic potential in HNSCC cell lines can be synergistically increased by co-treatment with paracetamol. A. Dose dependent cell viability assay in response to 96h DAC treatment in four HNSCC cell lines and normal oral keratinocytes (HOK) shows a bimodal HNSCC response to DAC. Grey box indicates clinically relevant concentrations. DAC-sensitive cell lines are shown in reds, DAC-resistant – in greys, HOKs – in blue. B. Global levels of DNA methylation (5mC) were analysed by DNA dot blot in HNSCC cell lines ± 500 nM DAC, 96h and show the demethylating DAC effect in DAC-sensitive cell lines only. The data were normalized to methylene blue staining and an example of 5mC dot blot is shown below. For each replicate the results for 1 μg and 0.5 μg of DNA were averaged. C. Paracetamol shows the largest DAC sensitizing effect in VU40T cells among 100 off patent drugs (Drug Library FMC1) used in the assay. The cells were treated with 500 nM DAC with or without one of the drugs and the cell viability was compared to the drug only control. The scatterplot shows the DAC sensitizing effect of each drug while the full data are included in Supplementary Fig. S1. Y axis represents increase in sensitivity, grey area shows ± 2SD. D. Cell viability in four HNSCC cell lines and HOK cells treated for 96h with 132.3 μM paracetamol (Para), 601.7 μM valproic acid (VPA) and 323.4 μM zinc acetate (Zinc) ±500 nM DAC. Only DAC-sensitive cell lines, VU40T and HN12, can be sensitized to DAC treatment by paracetamol. Dotted lines show the effect of 500 nM DAC alone. E-G. DAC-paracetamol synergy was determined using Chou-Talalay method. Cell viability data for VU40T (E) and HN12 (F) cells treated with fixed titrations of DAC C_max_ (500 nM), paracetamol C_max_ (132 μM) or DAC + paracetamol were used to calculate combination index (CI) (G) and dose reduction index (DRI) (Supplementary Tables S2 and S3). CI < 1 confirms synergistic interaction. In A-C and E-G n = 3, in D n = 3–7. In B statistical analysis was performed by paired two‐tailed *t* tests. In D, for each cell line a matched One-Way ANOVA with Dunnett's multiple comparison testing was used to compare DAC to DAC + drug. A separate paired *t*-test was applied to compare no treatment with DAC. Values are displayed as means ± SEM. Significant p values are shown. (For interpretation of the references to colour in this figure legend, the reader is referred to the Web version of this article.)

To establish the potential of DAC as a therapeutic in HNSCC, relative cell viability was initially determined in four HNSCC cell lines and in normal human oral keratinocytes (HOK) after 96h of treatment (Fig. 1A). HOK cells showed no decrease in viability at clinically relevant concentrations (IC_50_ of 8.93 μM) while the four HNSCC cell lines could be divided into two groups; DAC-sensitive (VU40T, IC_50_ of 2.17 μM and HN12, IC_50_ of 0.81 μM) and DAC-resistant (SCC040, IC_50_ of 10.61 μM and UDSCC2, non-responsive) (Fig. 1A). This pattern was mirrored in the ability of DAC to demethylate DNA in the sensitive cell lines only (Fig. 1B), suggesting the efficacy of DAC treatment is proportional to its ability to demethylate DNA.

### 3.2 Efficacy of DAC treatment can be synergistically increased with paracetamol

In patients with AML, a 5-day regimen of 20 mg/m^2^ DAC gave a maximum plasma concentration (C_max_) of 107 ng/ml, equivalent to 469 nM [6,28], while all HNSCC cell lines have an IC_50_ value greater than 500 nM. Therefore, one sensitive (VU40T) and one resistant (SCC040) cell line were subjected to a DAC sensitizing screen to establish whether the efficacy of DAC could be increased (Fig. 1C, Supplementary Fig. S1). In the DAC-resistant SCC040 cells, none of the drugs were able to sensitize the cells to DAC (Supplementary Fig. S1A). However, in the DAC-sensitive VU40T cell line, paracetamol, valproic acid (VPA) and zinc acetate further decreased cell viability (Fig. 1C, Supplementary Fig. S1B). The paracetamol effect was replicated in the DAC-sensitive HN12 but not in DAC-resistant UDSCC2 cells (Fig. 1D). Importantly, paracetamol alone did not alter the viability of HOK cells (Fig. 1D). Therefore, DAC + paracetamol combination was further tested for synergy in VU40T and HN12 cells using the Chou-Talalay method [22]. Cell viability was assessed in response to each drug separately and in combination across eight constant-ratio matched titration of C_max_ (500 nM for DAC and 132 μM for paracetamol) (Fig. 1E–F). This analysis showed combination index (CI) values less than 1 at all bar the lowest concentration, demonstrating synergy (Fig. 1G). In VU40T cells, the dose reduction index (DRI) indicated that, when used in combination, each drug can be reduced 5-fold (Supplementary Table S2), allowing DAC dose reduction from 2.26 μM to the clinically relevant 450 nM. A similar reduction was observed for HN12 cells (Supplementary Table S3).

### 3.3 Combined DAC + paracetamol treatment augments the effects of DAC on cell metabolism, proliferation and markers of basal epithelial cells

**Fig. 2:**
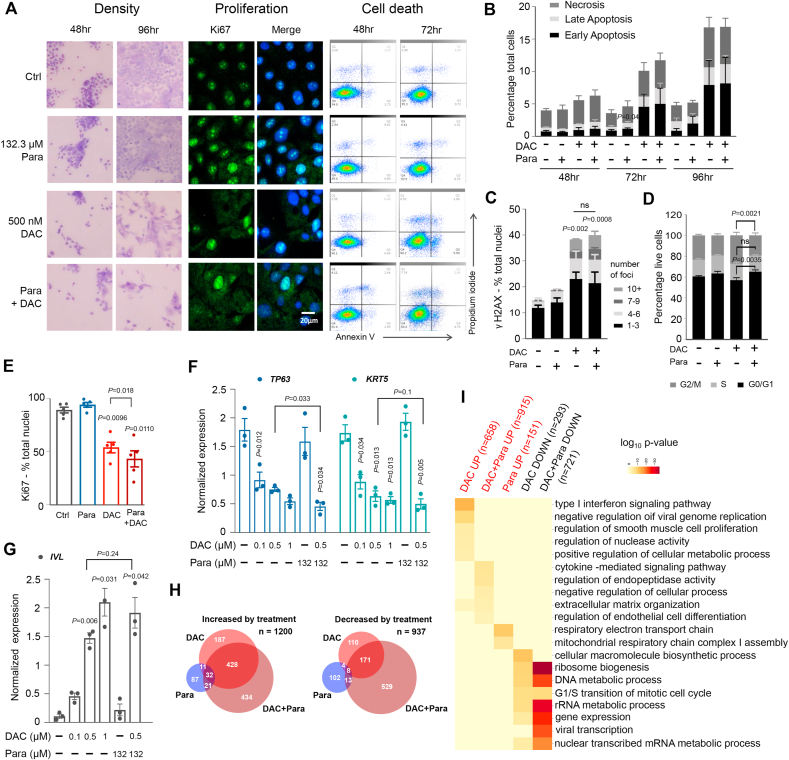
DAC-paracetamol combination enhances the effects of DAC on cell metabolism, proliferation and basal cell phenotype. A. Response of VU40T cells to paracetamol, DAC or both as indicated (representative images from 3 experiments). Left: Giemsa-Jenner staining (4x magnification); middle: Ki67 immunostaining; right: Annexin V and propidium iodine (PI) FACS analysis (healthy cells (bottom left); early apoptotic cells (bottom right); late apoptotic cells (top right) and necrotic cells (top left)). B. Proportion of VU40T cells undergoing cell death was assessed by FACS following Annexin V and PI staining (examples shown in (A)). The DAC-induced increase in cell death is not further enhanced by addition of paracetamol. C. Percentage of VU40T nuclei with indicated numbers of γH2AX foci following DAC, paracetamol and combined treatments. The DAC-triggered DNA damage is not enhanced by the addition of paracetamol. D. Cell cycle distribution in VU40T cells following 96h of indicated treatments and showing a slight increase in number of cells in G0/G1 phase following DAC + paracetamol treatment when compared to DAC alone. E. Addition of paracetamol to DAC treatment decreases proportion of nuclei positive for Ki67 immunostaining while paracetamol alone has no effect (examples in (A)). VU40T cells were treated as indicated for 96h. F. The expression of basal cell layer markers, *TP63* and *KRT5,* is down-regulated by DAC in a dose dependent manner; this is enhanced in DAC + paracetamol treatment while paracetamol alone has no effect. qRT-PCR results are shown as normalized to *ACTB* in VU40T cells treated as indicated. G. The expression of involucrin (*IVL,* marker of differentiated keratinocytes) is up-regulated by DAC in a dose dependent manner and this was more pronounced after addition of paracetamol. qRT-PCR results are shown as in (F). H. RNA-seq was performed in VU40T cells after 96h of DAC, paracetamol and DAC + paracetamol treatments. Venn diagrams show overlap for the most upregulated (left, log_2_ fold change ≥1) and down-regulated (right, log_2_ fold change ≤ -1) genes when compared to vehicle control. The number of genes affected by combined treatment is much higher than for DAC alone. Paracetamol treatment only moderately affects the cells’ transcriptome. I. Six gene sets (up-regulated or down-regulated by DAC, paracetamol and combined treatment) were analysed for enrichment of GO-Biological Processes pathways, further consolidated using REVIGO. Paracetamol treatment alone resulted in no significant enrichment for down-regulated gene sets. Top 5 terms from the remaining five groups are combined and included in the heatmap. Category scores are shown as log_10_ p-value. Unless stated otherwise all treatments were for 96h with 500 nM DAC and 132.2 μM paracetamol. In B-D and F-G n = 3, in E n = 6. In B and D: Two-Way ANOVA with Dunnett's correction was preformed to compare Ctrl with treatments and a separate Two-Way ANOVA with Sidak's was performed to compare DAC with combined treatment. In C: matched Two-Way ANOVA with Tukey's multiple comparison test was used to compare the distribution of foci number between each treatment group. In E-G: matched One-Way ANOVA with Dunnett's multiple comparison test was used to compare all treatments to Ctrl. A separate paired two-tailed *t*-test was used to compare DAC to DAC + paracetamol. Values are displayed as means ± SEM.

It was next investigated whether the mechanisms underlying the DAC + paracetamol synergy involved reduced proliferation, altered cell cycle progression or increased cell death, possibly due to enhanced DNA damage. DAC is known to cause cell death and DNA damage [2]. As expected, in VU40T, 500 nM DAC increased both apoptosis and necrosis (Fig. 2A–B) as well as the number of nuclei with γH2AX foci (a marker of DNA damage and double strand breaks) when compared to control (Fig. 2C); however, these were not additionally enhanced by the addition of paracetamol (Fig. 2A–C). When compared to untreated cells none of the treatments resulted in change in cell cycle progression, however, the addition of paracetamol to DAC led to fewer cells progressing to G2/M phase than for DAC alone (Fig. 2D). Similarly, combined treatment further decreased the number of Ki67-positive nuclei (a marker of proliferation) compared to DAC alone, whilst paracetamol alone had no effect (Fig. 2A, E). Markers for the basal cell layer (the only cell layer in healthy stratified epithelium that normally contains dividing cells), *TP63* and keratin 5 (*KRT5*), were down-regulated by DAC in a dose-dependent manner and this was enhanced by the addition of paracetamol (Fig. 2F). Conversely, involucrin (*IVL*), a marker for differentiated, suprabasal layers was upregulated upon DAC (Fig. 2G).

RNA sequencing of VU40T cells demonstrated substantial transcriptome alterations following DAC treatment and this was significantly enhanced by paracetamol (Fig. 2H). Six gene sets, up- and down-regulated by each treatment (Fig. 2H, Supplementary Table S4), were subjected to gene set enrichment analysis (Fig. 2I, Supplementary Table S5). Paracetamol treatment alone primarily resulted in up-regulation of genes involved in respiratory electron transport chain while the profile up-regulated by DAC was dominated by immune terms, especially interferon type I response. The latter was also evident in the combined treatment, although to a lesser extent. Interestingly, combined treatment was enriched for terms related to tissue development and differentiation (including ‘pharyngeal system development’, Supplementary Table S5). Genes down regulated by both DAC and DAC + paracetamol showed similar ontology groupings related to DNA, protein and RNA metabolism and this enrichment was much stronger for the combined treatment (Fig. 2I).

Therefore, although DAC alone has profound effects on proliferation, differentiation, cell death and DNA damage, the further reduction in viability observed in combined treatment is associated with a decrease in metabolism and proliferation, and divergence from the basal cell-like phenotype.

### 3.4 DAC treatment enhances the cyclooxygenase pathway, which is offset by paracetamol

**Fig. 3:**
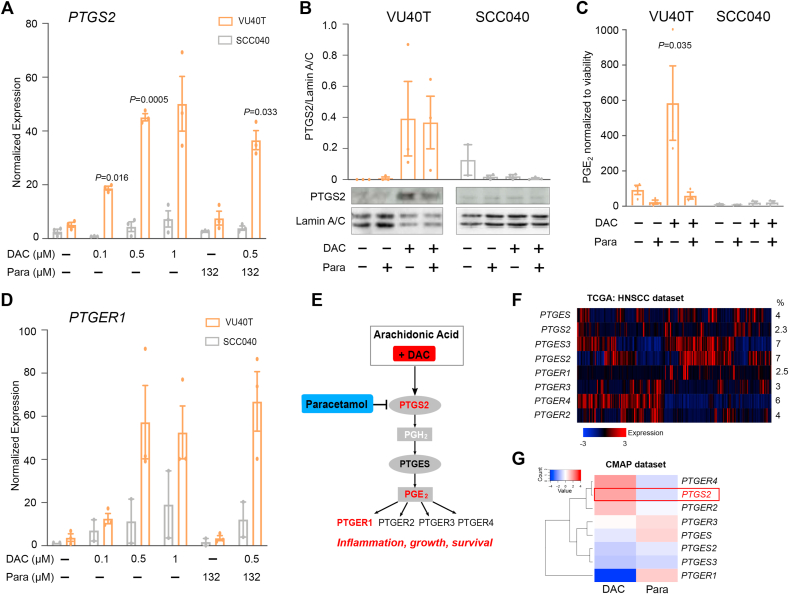
DAC treatment specifically activates COX-2-PGE_2_ pathway in DAC-sensitive cells. A. qRT-PCR for *PTGS2* shows increase in gene expression upon both DAC and DAC + paracetamol treatments in DAC-sensitive VU40T cells but not in DAC-resistant SCC040. The 96h treatments were performed as indicated. Results are normalized to cDNA concentration. B. PTGS2 protein levels are also up-regulated in VU40T but not in SCC040 cells. Graph represents the data from three experiments normalized to Lamin A/C. C. PGE_2_ concentration was assessed by ELISA in media collected from VU40T and SCC040 cells treated for 96h as indicated. The data are normalized to cell viability and show induction of PGE_2_ synthesis by DAC in DAC-sensitive VU40T cells only while the addition of paracetamol reverses this effect. D. qRT-PCR for PGE_2_ receptor, *PTGER1,* shown as in (A). DAC treatment, either alone or in combination with paracetamol, leads to an increase in *PTGER1* expression in VU40T cells while neither *PTGER1* nor *PTGER2-4* (Supplementary Fig. S2C) are up-regulated in DAC-resistant SCC040 cell line. E. Schematic of COX-2-PGE_2_ pathway with confirmed DAC effects shown in red. COX-2 catalyzes the conversion of arachidonic acid into prostaglandin H_2_. This is then converted into prostaglandin E_2_ which exerts its effects through G-protein coupled receptors. In this model DAC affects gene expression levels while paracetamol blocks protein function. The full COX-2 pathway is shown in Supplementary Fig. S2A. F. COX-2-PGE_2_ pathway-related gene expression across 522 HNSCC tumour samples indicates 150 (29%) of tumours have at least one of the genes up-regulated. However, *PTGS2* up-regulation is rare. Heatmap was created in cBioPortal based on provisional HNSCC cohort (TCGA). % indicates fraction of tumours with alterations for each of the genes. G. Expression of *PTGS2*, as well as *PTGER2* and *PTGER4,* is strongly up-regulated by DAC treatment in five cancer cell lines included in the BROAD Connectivity Map (CMAP) dataset which consists of transcriptional profiles following treatments with 1309 different drugs. Heatmap shows Drug Perturbation Signature for genes of the COX-2-PGE_2_ pathway for Decitabine (DAC) and paracetamol (Para). A-D: all treatments were for 96h with 500 nM DAC and 132.2 μM paracetamol and performed in three biological replicates. In A, B and D: for each cell line a matched One-Way ANOVA with Dunnett's multiple comparison test was used to compare all treatments to Ctrl; a separate paired two-tailed *t*-test was used to compare DAC to DAC + paracetamol. In C: for each cell line an ordinary One-Way ANOVA with Sidak's correction was applied to compare treated groups with Ctrl. Values are displayed as means ± SEM. Only significant p-values are shown. (For interpretation of the references to colour in this figure legend, the reader is referred to the Web version of this article.)

Paracetamol is understood to act on the cyclooxygenase pathway (Supplementary Fig. S2A), mainly through inhibition of COX-2 (PTGS2) [15]. Transcriptional activation of the COX-2-PGE_2_ pathway by DAC was also evident in the RNA-seq data (Supplementary Fig. S2B), and a geneset for ‘Prostanoid biosynthetic process’ was enriched after both DAC and combined treatments (Supplementary Table S5). Therefore, the effect of DAC on this pathway was further examined. In DAC-sensitive VU40T cells but not DAC-resistant SCC040 cells, *PTGS2* RNA and protein levels were upregulated by DAC and combined treatment (Fig. 3A and B). A corresponding increase in the downstream product, prostaglandin E_2_ (PGE_2_) was observed after DAC treatment and returned to basal levels by the addition of paracetamol (Fig. 3C). This indicates that paracetamol can diminish DAC-induced COX-2 pathway activation. Additionally, expression of the PGE_2_ receptor *PTGER1* increased in the DAC-sensitive VU40T cells whilst no significant changes in *PTGER1-4* expression occurred in SCC040 (Fig. 3D, Supplementary Fig. S2C). The up-regulation of *PTGS2* and *PTGER1-2* can also be observed in HN12 cells; although the changes are less pronounced, they are highly consistent across DAC titrations (Supplementary Fig. S2D). There is a possibility that blocking the COX pathway could shunt AA towards the lipoxygenase (LOX) pathway, leading to increased survival potential [29]; however, we did not find evidence of this in DAC + paracetamol-treated HNSCC cells (Supplementary Fig. S3). In summary, DAC treatment specifically upregulates many aspects of the COX-2-PGE_2_ pathway, inadvertently providing the cancer cells with growth and survival potential, while the addition of paracetamol offsets this effect (Fig. 3E).

### 3.5 DAC treatment complements cancer-related activation of COX-2-PGE_2_ pathway

Activation of the COX-2-PGE_2_ pathway has been previously indicated in both HNSCC and other cancer types [16,17]. In the TCGA cohort, 29% of HNSCC tumours have at least one component of COX-2-PGE_2_ pathway transcriptionally activated, mostly PGE_2_ synthases or PGE_2_ receptors (Fig. 3F). Similar activation can be observed in other cancers (Supplementary Table S6). However, over-expression of *PTGS2* itself is relatively rare (2.3%), potentially serving as a limiting factor in the full pathway activation. Therefore, DAC-induced *PTGS2* up-regulation could remove this limitation and counteract the anti-tumour effects of DAC. *PTGS2* up-regulation by DAC was also detected using Drug Perturbation Signatures (Fig. 3G) in the cMAP dataset [30], indicating the potential of DAC + paracetamol co-treatment could be applicable to other tumour types.

### 3.6 Combined treatment depletes glutathione levels and increases oxidative stress in HNSCC cells

**Fig. 4:**
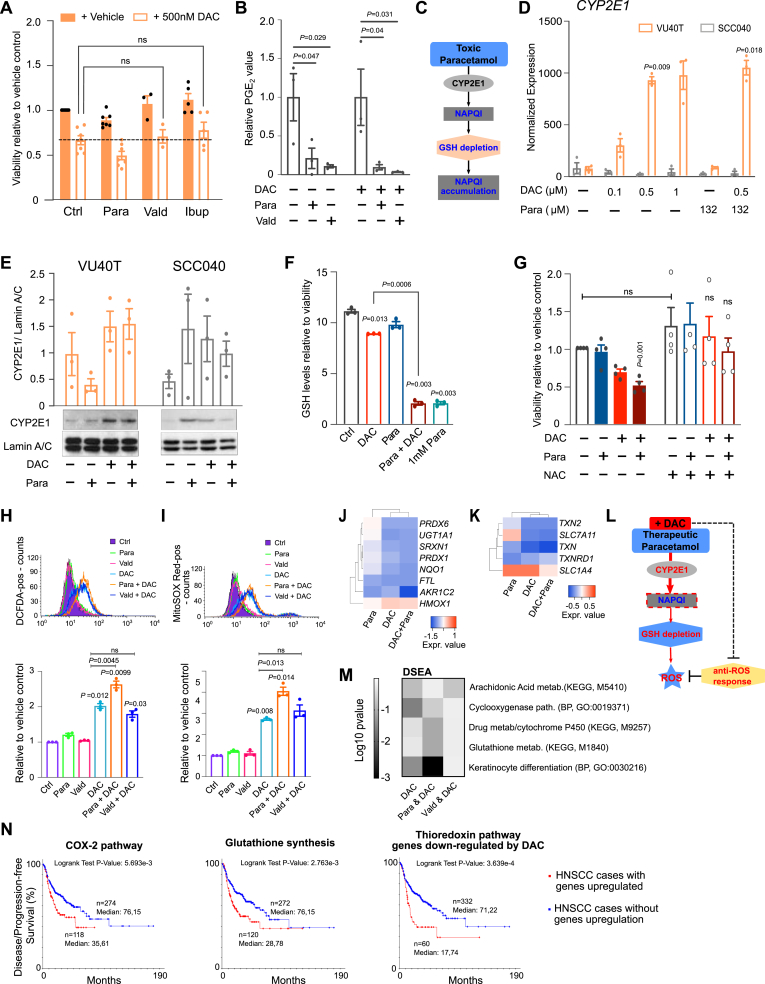
Combined DAC-paracetamol treatment mimics the effects of paracetamol overdose, depletes glutathione levels and leads to oxidative stress. A. Cell viability in VU40T cells treated for 96h with 132.3 μM paracetamol, 10 μM valdecoxib or 193.9 μM ibuprofen ± 500 nM DAC. Neither ibuprofen nor valdecoxib treatment could reproduce the DAC-sensitizing effect observed for paracetamol, indicating the effect is paracetamol-specific. Dotted line shows the effect of DAC alone. **B.** PGE_2_ concentration in the media of VU40T cells treated with DAC with or without paracetamol or valdecoxib shown as relative to either vehicle control (left) or DAC only treatment control (right). Valdecoxib blocked PGE_2_ synthesis in both DAC-treated and untreated cells, comparably to paracetamol, suggesting synergistic effects act outside of the COX2-PGE_2_ pathway. C. Schematic of paracetamol overdose. A fraction of paracetamol is converted into the toxic metabolite NAPQI, and detoxified by GSH. However, when paracetamol is taken in excess (toxic dose) GSH stores deplete and NAPQI accumulates. D. qRT-PCR for *CYP2E1* shows increase in gene expression upon both DAC and DAC + paracetamol treatments in DAC-sensitive VU40T cells but not in DAC-resistant SCC040. The 96h treatments were performed as indicated and the results are shown normalized to the cDNA concentration. E. CYP2E1 protein levels in VU40T and SCC040 cells also show increase in VU40T cells only. The graph represents the data from three experiments normalized to Lamin A/C. F. Glutathione (GSH) levels are reduced significantly in cells treated with DAC + paracetamol as compared to DAC alone. VU40T cells were treated for 96h with DAC, paracetamol or both and the results were normalized to cell viability. 1 mM paracetamol was used as a positive control representing toxic concentrations. G. NAC rescue restores cell viability in cells treated with both DAC and DAC + paracetamol. VU40T cells were pre-treated for 48h with 2.5 mM NAC followed by 96h of DAC, paracetamol or combined treatments (right panel) and cell viability was assessed in comparison to control without NAC (left panel). H. Combined treatment significantly enhances oxidative stress when compared to DAC alone; a paracetamol specific effect, not observed in co-treatment with valdecoxib. Levels of intracellular ROS (DCFDA staining and FACS) in VU40T cells treated for 72h as indicated. Upper figure: representative FACS profile; lower graph: geometric mean normalized to vehicle control. I. The exacerbation of oxidative stress in combined treatment was also detected by increased levels of mitochondrial superoxide (MitoSOX Red staining) assessed as in (H). J. Gene expression changes of direct and known responders to oxidative stress were extracted from the VU40T RNA-seq data set and show common down-regulation following DAC and DAC + paracetamol treatments. They are represented as heatmap of log_2_ fold change values after indicated treatments. K. Genes implicated in maintaining intracellular cysteine pools (uptake of cystine by SLC7A11 or cysteine by SLC1A4) and in thioredoxin reductase system (TXN/TXNRD) are also down-regulated by DAC and DAC + paracetamol treatment (shown as in (J)). L. Schematic of proposed mechanism underlying DAC-paracetamol synergy: mimicry of paracetamol overdose through DAC-induced up-regulation of *CYP2E1*, which in the presence of paracetamol leads to GSH depletion and exacerbation of oxidative stress. The effects of DAC are shown in red and paracetamol contribution – in blue. M. Drug Set Enrichment Analysis was used to identify the mechanisms of action shared by DAC + paracetamol combination and compared to DAC + valdecoxib and DAC alone. Log_10_ p-values for selected pathways of interest (KEGG or GO BP genesets) are shown as heatmap. N. Disease/Progression-free survival curves for 392 HNSCC patients with data available in TCGA provisional cohort with or without upregulation of genes (z-score ≥2.0) implicated in pathways behind DAC-paracetamol drug interaction (for gene sets see Supplementary Methods). Kaplan Meier Estimates were plotted using cBioPortal. Left: Up-regulation of genes involved in COX-2-PGE_2_ pathway correlates with decreased survival. Middle: A negative effect on patients' survival is observed when genes involved in glutathione synthesis are up-regulated, in agreement with a proposed scenario wherein maintaining GSH stores is required for cancer cell survival. Right: A poorer survival is observed when genes involved in thioredoxin responses are up-regulated, enabling cellular adaptation to oxidative stress and protection from oxidative damage. Unless stated otherwise all treatments were for 96h with 500 nM DAC and 132.2 μM paracetamol, n = 3 for A-B, D-F, H–I and n = 4 for G. In A: a mixed -effect analysis with Dunnett's correction was used to compare DAC to DAC + Vald and DAC + Ibup samples. In B, D–F: One-Way ANOVA with Dunnett's correction was used to compare treatments with Ctrl. In D and F: a separate paired two-tailed *t*-test was used to compare DAC to DAC + paracetamol. In G: for each group (+vehicle, +NAC) a matched One-Way ANOVA with Dunnett's was used to compare treatments with the respective Ctrl; additionally, a paired two-tailed *t*-test was used to compare Ctrl with NAC. In H–I: a matched One-Way ANOVA with Dunnett's correction was used to compare all groups to Ctrl; a separate ANOVA was used to compare DAC to DAC + Para and DAC + Vald. Values are displayed as means ± SEM. Only significant p values are shown. (For interpretation of the references to colour in this figure legend, the reader is referred to the Web version of this article.)

If COX-2-PGE_2_ pathway alterations were solely responsible for DAC-paracetamol synergy, other COX inhibitors should have a similar effect. However, neither ibuprofen, nor the COX-2-specific inhibitor valdecoxib sensitized HNSCC cells to DAC treatment (Fig. 4A, Supplementary Fig. S4A-B). This was evident despite valdecoxib being able to reduce DAC-stimulated PGE_2_ synthesis comparably to paracetamol (Fig. 4B) and suggested an alternative mechanism.

Previous work on the anti-cancer therapeutic potential of paracetamol involved toxic doses, with efficacy attributed to an accumulation of the toxic metabolite of paracetamol, N-acetyl-p-benzoquinone-imine (NAPQI), resulting in glutathione depletion [[19], [20], [21]] (Fig. 4C). By comparison, our current work was performed using a safe concentration of paracetamol, 132 μM. However, in DAC-sensitive VU40T cells, DAC treatment caused an up-regulation of the majority of CYP enzymes-encoding genes (Supplementary Fig. S5A), including *CYP2E1*, thought to be primarily involved in the conversion of paracetamol into NAPQI (Fig. 4D–E). Assessing NAPQI levels is unreliable due to its highly reactive nature [31]. However, combined treatment led to a major reduction in GSH levels, a surrogate marker often used, and the effect was equivalent to high dose (1 mM) paracetamol (Fig. 4F). Furthermore, 48h pre-treatment with 2.5 mM N-acetyl cysteine (NAC, used clinically as an antidote to paracetamol overdose [19]) restored the viability of DAC + paracetamol treated cells to control level (Fig. 4G). Therefore, the combined treatment leads to the depletion of GSH stores in tumour cells, even at a clinically safe paracetamol concentration.

GSH is an intracellular antioxidant that acts as a reactive oxygen species (ROS) scavenger [32]. In agreement with this, DAC + paracetamol co-treatment significantly increased both intracellular ROS (Fig. 4H) and mitochondrial superoxide (Fig. 4I) when compared to DAC alone. This was specific for DAC + paracetamol and not DAC + valdecoxib (Fig. 4H–I). The effects of combined treatment on GSH, ROS and mitochondrial superoxide levels were also observed in HN12 cells (Supplementary Fig. S5B-E). Accumulation of ROS triggers an anti-oxidant response which can protect cancer cells treated with chemotherapeutics [33]. However, the RNA-seq data for VU40T cells showed the majority of genes described as direct anti-oxidant responders [34] to be down-regulated upon both DAC and DAC + paracetamol (Fig. 4J), potentially exacerbating oxidative stress. Finally, it has been shown that keratinocytes can tolerate GSH depletion as long as the cysteine pools and the thioredoxin reductase system (TXN/TXNRD) are functional [35]. Again, DAC down-regulated most of these genes in VU40T cells (Fig. 4K).

To summarize, in cancer cells, DAC-paracetamol co-treatment mimics the mechanisms of paracetamol overdose (Fig. 4L), whilst cell adaptation to oxidative stress could be impaired by DAC, therefore improving the efficacy of anti-cancer drugs [36].

The specificity of the DAC-paracetamol interaction is further supported through Drug Set Enrichment Analysis (DSEA) [27] which identified that DAC and paracetamol share significant enrichment not only for COX, cytochrome P450 and GSH metabolism pathways, but also for keratinocyte differentiation. Such common enrichment was not observed for DAC and valdecoxib combination (Fig. 4M, Supplementary Fig. S6). Importantly, the expression of gene sets identified behind the DAC and paracetamol interaction (namely, suppression of DAC-activated COX-2-PGE_2_ pathway, depletion of the GSH stores and thioredoxin response) significantly affects survival in HNSCC (Fig. 4N and Supplementary Table S6) and other cancers (Supplementary Table S7) in TCGA data sets. This confirms that the DAC + paracetamol combination targets pathways and genes of clinical importance.

### 3.7 *In vivo* potential of DAC + paracetamol combination treatment

**Fig. 5:**
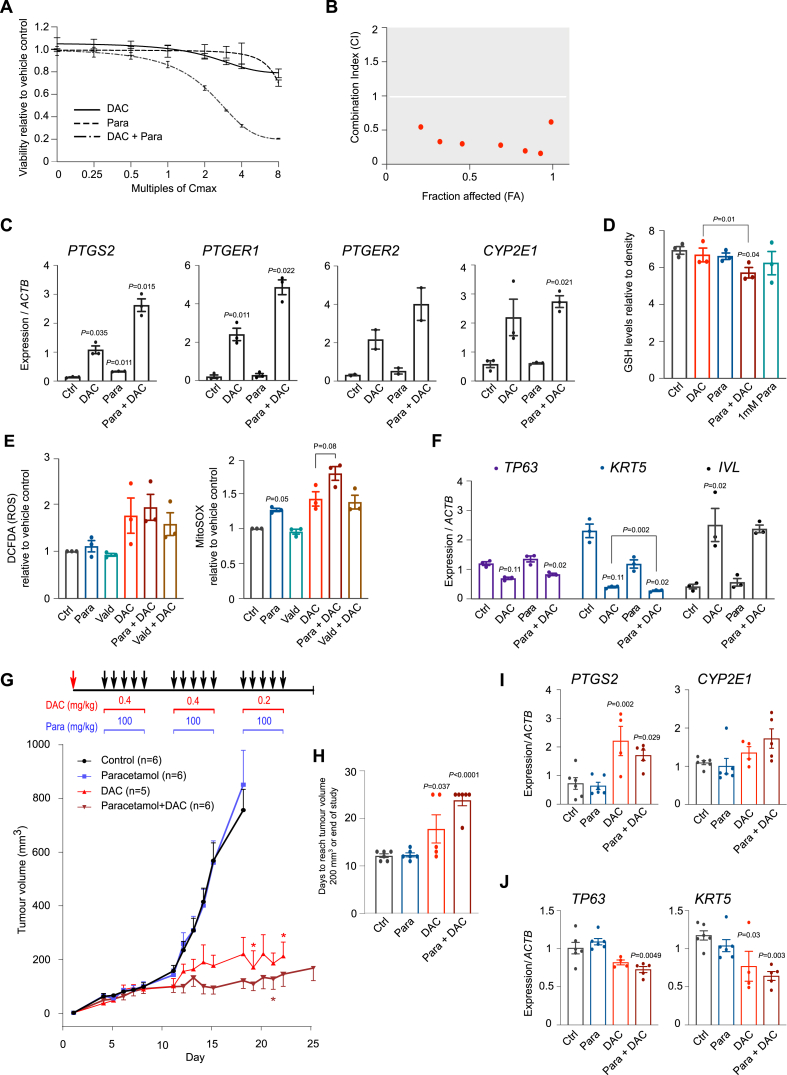
*In vivo* potential of DAC + paracetamol combined treatment in HNSCC. A-B. DAC-paracetamol synergy was confirmed in HNSCC FaDu cells prior to their use in mice xenografts (shown as in Fig. 1E–G); cell viability was assessed after treatment with fixed C_max_ titrations of the drugs (A). The resulting combination index (CI) is below 1 for all concentrations, indicating synergy (B). C. Gene expression of *PTGS2*, its receptors (*PTGER1* and *PTGER2*) and *CYP2E1* increase in FaDu cells following DAC and DAC + paracetamol treatments, in agreement with the data presented in Fig. 3, Fig. 4. qRT-PCR data are shown relative to *ACTB*. D. Glutathione (GSH) levels are reduced significantly in cells treated with DAC + paracetamol as compared to DAC alone. FaDu cells were treated for 96h with DAC, paracetamol or both and the results were normalized to cell density. 1 mM paracetamol was used as a positive control representing toxic concentrations. E. Combined treatment increases the levels of ROS (left) and, significantly, mitochondrial superoxide (right) when compared to DAC alone; a paracetamol specific effect, not observed in co-treatment with valdecoxib. Levels of intracellular ROS (DCFDA staining and FACS) and mitochondrial superoxide (MitoSOX Red staining) assessed as in (Fig. 4H–I).F. The expression of markers of the basal cell layer (*TP63* and *KRT5)* and differentiated keratinocytes (*IVL*) upon DAC and paracetamol treatment in FaDu cells. qRT-PCR results are shown as normalized to *ACTB*. G. Tumour growth of FaDu cells engrafted into NSG mice (Day 1, red arrow) and treated as indicated (black arrows) suggests the efficacy of DAC treatment is enhanced *in vivo* by co-treatment with paracetamol. * indicates mouse culling due to disease progression. H. Days to reach tumour size of 200 mm^3^ in each treatment. Tumours below 200 mm^3^ at the end of the experiment are counted as Day 25. I. qRT-PCR performed on RNA extracted from tumour tissues shows DAC-induced up-regulation of *PTGS2* and *CYP2E1,* confirming the *in vitro* results. Results are normalized to *ACTB*. J. qRT-PCR performed on RNA extracted from tumour tissues shows *TP63* and *KRT5* are down-regulated by DAC, with stronger effect observed in combined treatment in agreement with the *in vitro* data. Results are normalized to *ACTB*. In A-F n = 3. In G-H: Ctrl (n = 6), paracetamol (n = 6), DAC (n = 5), DAC + paracetamol (n = 6). In I-J: Ctrl (n = 6), paracetamol (n = 6), DAC (n = 4), DAC + paracetamol (n = 5). C,D,F: for each group a matched One-Way ANOVA with Dunnett's was used to compare treatments with Ctrls; additionally, a paired two-tailed *t*-test was used to compare DAC with DAC + Para. In E: a matched One-Way ANOVA with Dunnett's correction was used to compare all groups to Ctrl; a separate ANOVA was used to compare DAC to DAC + Para and DAC + Vald. In H-J: a non-matched One-Way ANOVA with Dunnett's correction was used to compare treatments with control. To compare DAC and DAC + Para an unpaired two‐tailed *t*-test was used. Values are displayed as means ± SEM. Only significant p values are shown.

To assess the DAC + paracetamol treatment *in vivo,* we utilized an NSG mouse xenograft model using human HNSCC FaDu cells [24]. FaDu cells were first confirmed *in vitro* to respond synergistically to DAC-paracetamol co-treatment (Fig. 5A–B). Similar to what was observed in VU40T cells, DAC treatment up-regulated expression of *PTGS2* and *PTGER1-2* (Fig. 5C). In addition, *CYP2E1* expression increased significantly upon DAC treatment (Fig. 5C) while combined treatment led to enhanced GSH depletion (Fig. 5D) and mitochondrial superoxide accumulation (Fig. 5E). The thioredoxin and anti-oxidant response genes were also slightly down-regulated (Supplementary Fig. S7). In further agreement with the VU40T data the DAC-imposed down-regulation of basal cell marker *KRT5* was significantly enhanced by the combined treatment (Fig. 5F).

Mice were injected in the right flank and the treatments (DAC, paracetamol, DAC + paracetamol or vehicle (PBS)) were administered 5 days a week (Fig. 5G). After the first two weeks, tumours in the control and paracetamol-treated groups reached the maximum permissible size, while DAC alone and DAC + paracetamol groups were treated for another week (Fig. 5G). Due to the strong initial response to DAC, the concentration was reduced (0.2 mg/kg) for the final week. Although DAC alone showed a strong anti-tumour effect, the DAC + paracetamol-treated tumours remained consistently smaller throughout the treatment, no tumours exceeded 300 mm^3^ and 5/6 animals survived until the end of the experiment (Fig. 5G–H). Furthermore, the gene expression alterations in tumour tissues were consistent with the *in vitro* observations: upregulation of *PTGS2* and *CYP2E1* (Fig. 5I) and down-regulation of *TP63* and *KRT5* (Fig. 5J). In summary, the *in vivo* data support the potential use of DAC in the treatment of DAC-sensitive HNSCC tumours. They also suggest paracetamol could increase DAC efficacy, however, due to a very strong reaction to DAC alone at 0.4 mg/kg, we were unable to detect a statistically significant decrease in tumour size upon the addition of paracetamol. Therefore further investigations, using lower DAC dose from the start of treatment, are needed to confirm the benefits of combined treatment *in vivo*.

### 3.8 Synergistic effects of DAC + paracetamol co-treatment are also observed in AML cell lines

**Fig. 6:**
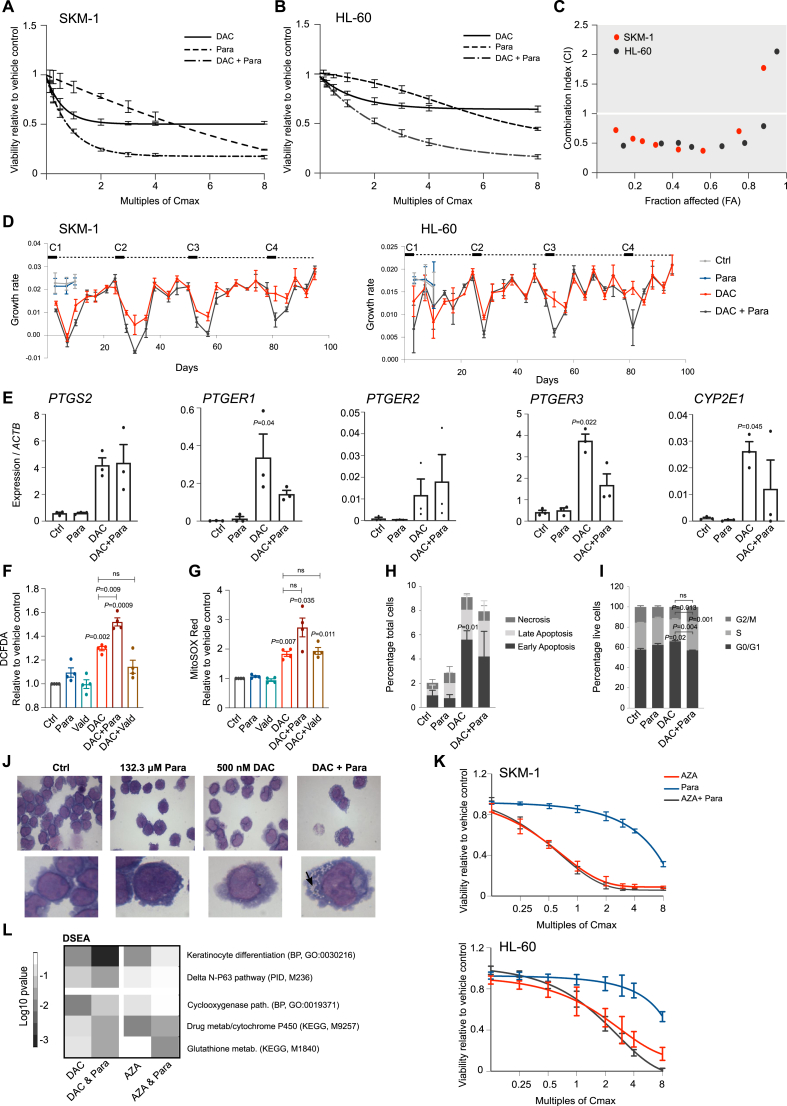
Potential of DAC-paracetamol combined treatment in AML. A-C. DAC-paracetamol synergy was established in AML cell lines, SKM-1 (A) and HL-60 (B), treated as indicated for 72h. Cell viability data were used to calculate combination index (CI) which confirmed synergy in both (C). D. Long term effects of DAC and DAC-paracetamol combined treatment on growth rates were assessed in SKM-1 (left) and HL-60 (right) cells over four treatment cycles (C1–C4, 72h treatment as indicated followed by 21-day withdrawal), indicating paracetamol could prolong the efficacy of DAC treatment. Vehicle and paracetamol controls are shown for the first 10 days. E. Gene expression of *PTGS2*, its receptors (*PTGER1*, *PTGER2* and *PTGER3*) and *CYP2E1* increase in SKM-1 cells following DAC and DAC + paracetamol treatments in agreement with the HNSCC data. qRT-PCR data are shown normalized against *ACTB*. F. Levels of intracellular ROS measured by DCFDA staining and FACS in SKM-1 cells treated for 72h with indicated drugs and their combinations. Vald, 10 μM valdecoxib. The ROS levels are increased in DAC + paracetamol, but not DAC + valdecoxib, when compared to DAC alone in agreement with the HNSCC data. The results are shown as geometric mean normalized to vehicle control. G. Levels of mitochondrial superoxide assessed by MitoSOX Red staining and FACS in SKM-1 cells as in (F) also show that only the addition of paracetamol exacerbates oxidative stress in DAC-treated cells. H. Proportion of SKM-1 cells undergoing cell death was assessed by FACS following Annexin V and PI staining. The DAC-induced increase in cell death is not additionally enhanced by addition of paracetamol. I. Cell cycle distribution of SKM-1 cells following 72h of indicated treatments. J. Giemsa-Jenner staining in SKM-1 cells reveals enhanced features of myeloid differentiation after combined treatment, increased cytoplasm with vacuole formation (black arrow). Upper: 100x magnification; lower: image zoomed to approximately one cell. K. Cell viability experiments in SKM-1(upper graph) and HL-60 (lower graph) cells after titrations of the C_max_ of AZA (3 μM), paracetamol (132.2 μM) or both for 72h. Results indicate that paracetamol does not sensitize cells to 5-azacitidine treatment. (For interpretation of the references to colour in this figure legend, the reader is referred to the Web version of this article.) L. DSEA was used to identify the differences in the mechanisms of action between DAC and azacitidine (AZA) and their combinations with paracetamol. While ‘Keratinocyte differentiation’ and ‘TP63 pathway’ are among the most enriched terms for DAC + paracetamol combination, they are not enhanced by AZA + paracetamol. In addition, AZA does not activate the cyclooxygenase pathway to the same extend as DAC; and cytochrome P450 metabolism term is only enhanced by addition of paracetamol to DAC but not AZA. Log_10_ p-values for selected pathways of interest are shown as heatmap. Unless stated otherwise all treatments were for 72h with 500 nM DAC and 132.2 μM paracetamol and n = 3. In E-G: a matched One-Way ANOVA with Dunnett's correction was applied to compare treatments with control; in F-G a separate ANOVA was used to compare DAC to DAC + Para and DAC + Vald. In H–I: for each group a matched Two-Way ANOVA with Dunnett's was performed to compare treatments with control; a separate Two-Way ANOVA with Sidak's was used to compare DAC with DAC + Para. Values are displayed as means ± SEM. Only significant p values are shown.

DAC currently has EMA approval for the treatment of AML [6]. Therefore, combined treatment of DAC + paracetamol was tested in two AML cell lines, SKM-1 and HL-60, and the drugs were found to work synergistically in both (Fig. 6A–C). The combined effect was even more apparent when the DAC treatment regimen used in AML patients was mimicked [28] (72h treatment, 21 days withdrawal, four cycles, Fig. 6D); by the fourth cycle the cells were still responding to combined treatment while becoming resistant to DAC alone (Fig. 6D).

Similar to HNSCC cells, DAC treatment up-regulated *PTGS2* and PGE_2_ receptors’ expression in both AML cell lines, and *CYP2E1* expression in SKM-1 cells (Fig. 6E, Supplementary Fig. S8A). Ultimately, DAC increased ROS and mitochondrial superoxide production in these cells and this was significantly enhanced by the addition of paracetamol, but not valdecoxib (Fig. 6F–G).

Furthermore, combined treatment did not add to the cell death caused by DAC alone (Fig. 6H) and resulted only in a slight increase in number of cells retained in S phase when compared to DAC alone (Fig. 6I). A change towards myelocyte morphology (increased cytoplasm and vacuoles’ numbers) was most apparent after DAC + paracetamol combined treatment (Fig. 6J), although the expression of myeloid differentiation marker *CD11b* (*ITGAM*) was similarly up-regulated upon DAC and combined treatments (Supplementary Fig. S8B). These data indicate that DAC-paracetamol synergy and the effect it has on oxidative stress could be applicable to blood malignancies, where DAC is an accepted therapeutic option. 5-azacitidine (AZA) is another DNA methylation inhibitor approved in the treatment of AML and myelodysplastic syndrome (MDS) [37,38]. Despite both drugs acting by replacing cytosines in the DNA, their effects on transcription and metabolism differ significantly [39] which is believed to be caused by 5-azacitidine, but not DAC, being preferentially incorporated into RNA [2,39]. Here, we show that paracetamol has no effect on 5-azacitidine treatment in either SKM-1 or HL-60 cells (Fig. 6K). A DSEA investigation indicated disparate impact of 5-azacytidine and paracetamol on keratinocyte differentiation, cyclooxygenase and P450 metabolism pathways in comparison to DAC and paracetamol (Fig. 6L).

Therefore, the synergistic effect of paracetamol is specific to co-treatment with DAC.

## 4 Discussion

The search for new drugs to improve the survival rate of HNSCC is ongoing. Our results using four HNSCC cell lines and an *in vivo* mouse model show that DAC alone has therapeutic potential in HNSCC. However, the response is variable, as has been observed for other solid tumours [8] and patient stratification using predictive biomarkers will be necessary to identify the DAC-responders. The results shown here demonstrate that DAC sensitivity is primarily dependent upon the drug's ability to demethylate DNA, and therefore likely due to incorporation, activation or retention of the drug, as suggested previously [40,41]. Furthermore, an initial response to DAC was a prerequisite for synergy with paracetamol, hence the co-treatment is dependent upon the DNA demethylating capacity of DAC.

Paracetamol is routinely prescribed as an analgesic, however so far it has not been considered whether its use may influence the efficacy of chemotherapy regimens. Our study demonstrates that paracetamol can enhance the anti-tumour activity of DAC with two main translational impacts. Adding paracetamol to DAC treatment could significantly lower the DAC dose needed to achieve therapeutic effects, potentially reducing DNA damage-related side effects and ultimately broadening DAC application. It also highlights that uncontrolled use of paracetamol during cancer therapies and clinical trials could affect the outcomes and interpretation of the results. Hence, further studies are required to look at the impact of supportive care medication in oncology.

**Fig. 7:**
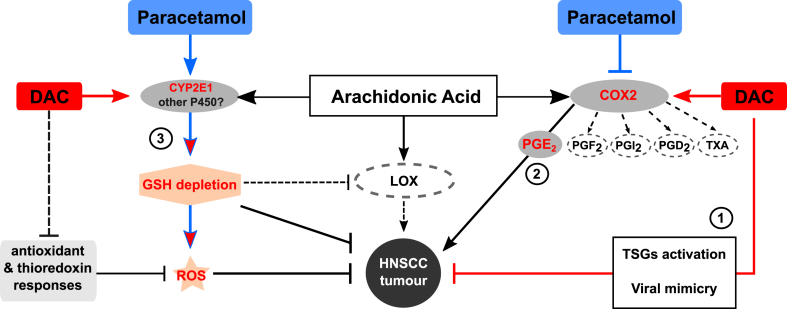
Mechanisms underlying DAC-paracetamol interaction. Arachidonic acid (AA) is metabolized to eicosanoids through COX, LOX, and cytochrome P450 monooxygenase pathways. In addition to DAC limiting cancer cell growth through either activation of tumour suppressor genes (TSGs) or viral mimicry (1), it inadvertently activates COX-2 PGE_2_ pathway (2), which is contradicted by paracetamol. DAC also upregulates *CYP2E1* which, in the presence of paracetamol, leads to glutathione depletion and ROS accumulation, both enhanced by combined treatment (3). Our preliminary data also indicate that DAC potentially downregulates transcription of genes involved in antioxidant and thioredoxin responses, preventing cancer cells from developing adaptation to oxidative stress and protection from oxidative damage. In addition, GSH depletion has the potential to limit the production of LOX pathway metabolites dependent on GSH transferases.

In this report we identified key mechanisms within the AA metabolism pathway that could underlie the synergy between DAC and paracetamol (Fig. 7). Both our data and analysis of public databases point towards DAC explicitly upregulating COX-2-PGE_2_ pathway, *PTGS2* in particular, potentially providing cancer cells with survival advantage. However, it remains to be established whether this is a direct result of DNA demethylation or is rather due to indirect mechanisms (e.g. response to dsRNA, cytokines or growth factors).

Surprisingly, the synergistic effect observed for paracetamol could not be reproduced using other COX-2 inhibitors. Although it is possible some tumour cells could still be affected by the suppression of COX-2-PGE_2_ pathway, the data indicate an alternative and paracetamol-specific mechanism; DAC-induced mimicry of paracetamol overdose, leading to GSH depletion and exacerbated oxidative stress, both of which have the potential to restrict tumour growth and improve patient survival [36]. The synergistic effect was also specific to DAC and not applicable to the structurally similar DNA methylation inhibitor, 5-azacitidine. The mechanisms described in this study likely explain the specificity of DAC-paracetamol drug interaction.

Response to DAC has a profound effect on the transcriptional programme in HNSCC cells and is dominated by activation of type I interferon and anti-viral pathways, agreeing with recent reports on the role of ‘viral mimicry’ in cancer treatment with DNA demethylating agents [3,4]. These effects are maintained but not increased by combined treatment and, since the immune response is currently thought to be an important part of response to DAC, it would be beneficial to test the synergy between DAC and paracetamol in an immune competent *in vivo* model.

Instead, the addition of paracetamol to DAC treatment led to a decrease in DNA, RNA and protein metabolism, together with reduced proliferation and enhanced differentiation. Notably, ‘keratinocyte differentiation’ emerged in the DSEA as one of the most enriched terms affected specifically by DAC-paracetamol combination. Further experiments are required to establish the exact mechanisms leading to the changes in cell proliferation and differentiation upon combined treatment. One possibility involves the effects of AA metabolism and ROS on PI3K/Akt and MAPK pathways, the main proliferation drivers in HNSCC [42].

This study demonstrates that the commonly used drug, paracetamol, available as over-the-counter medicine and often self-medicated by patients, can change the cancer cell response to a chemotherapeutic. Therefore, considering the mechanisms described here, paracetamol interaction with other drugs, especially chemotherapeutics, should be taken into consideration. This manuscript provides a solid rationale for the controlled use of paracetamol in AML, where DAC treatment has already been approved and suggests efficacy may also be applicable to HNSCC. Since paracetamol is a very cheap and relatively safe drug, it could be added to treatment with minimal cost but considerable translational impact.

## Declaration of competing interest

The authors declare no conflict of interest.
