# Supplementary material for: The anti-tumour activity of DNA methylation inhibitor 5-aza-2′-deoxycytidine is enhanced by the common analgesic paracetamol through induction of oxidative stress
Source: Cancer Lett. 2021 Mar 31;501:172–86. doi: 10.1016/j.canlet.2020.12.029 (PMC7845757; doi:10.1016/j.canlet.2020.12.029)
Supplement: The following are the supplementary data related to this article:Multimedia component 1 [file mmc1.pdf]

A

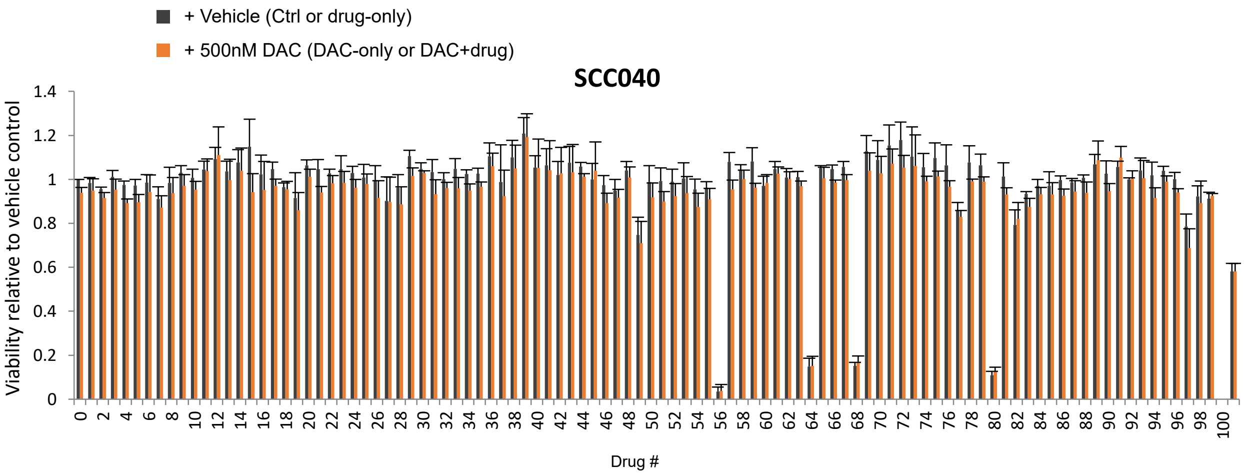

B

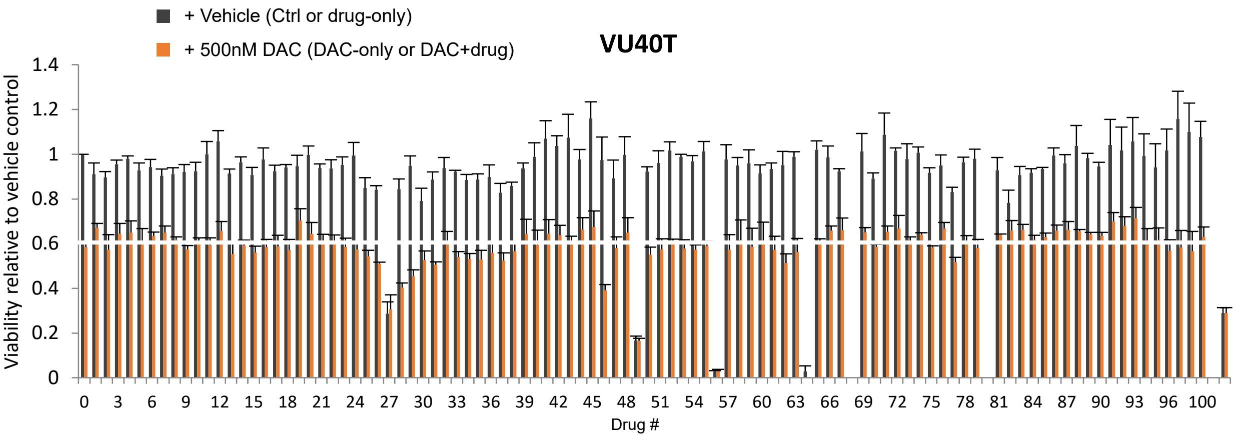

Figure S1. Sensitivity of HNSCC cells to DAC treatment can be increased by drug combinations (related to Fig. 1).

A-B. DAC sensitizing assay: DAC-resistant SCC040 (**A**) and DAC-sensitive VU40T (**B**) cells were subjected to 96h treatment with one of a panel of 100 drugs (Drug Library FMC1), with or without 500 nM DAC. Viability was recorded and is shown here relative to the vehicle only control cells (Drug #0 black bar). The horizontal white line shows the effect of 500 nM DAC alone. The bars far right (Drug #101) show the effect of 10 µM DAC. The sensitizing effect is observed when the combined effect of the two drugs is more effective than both DAC alone and the drug alone. The assay was performed in triplicate and error bars represent SEM.

Drugs with sensitizing effect: #28 - zinc acetate, #29 - valproic acid, #46 - paracetamol.
