# Supplementary material for: The anti-tumour activity of DNA methylation inhibitor 5-aza-2′-deoxycytidine is enhanced by the common analgesic paracetamol through induction of oxidative stress
Source: Cancer Lett. 2021 Mar 31;501:172–86. doi: 10.1016/j.canlet.2020.12.029 (PMC7845757; doi:10.1016/j.canlet.2020.12.029)
Supplement: Multimedia component 2 [file mmc2.pdf]

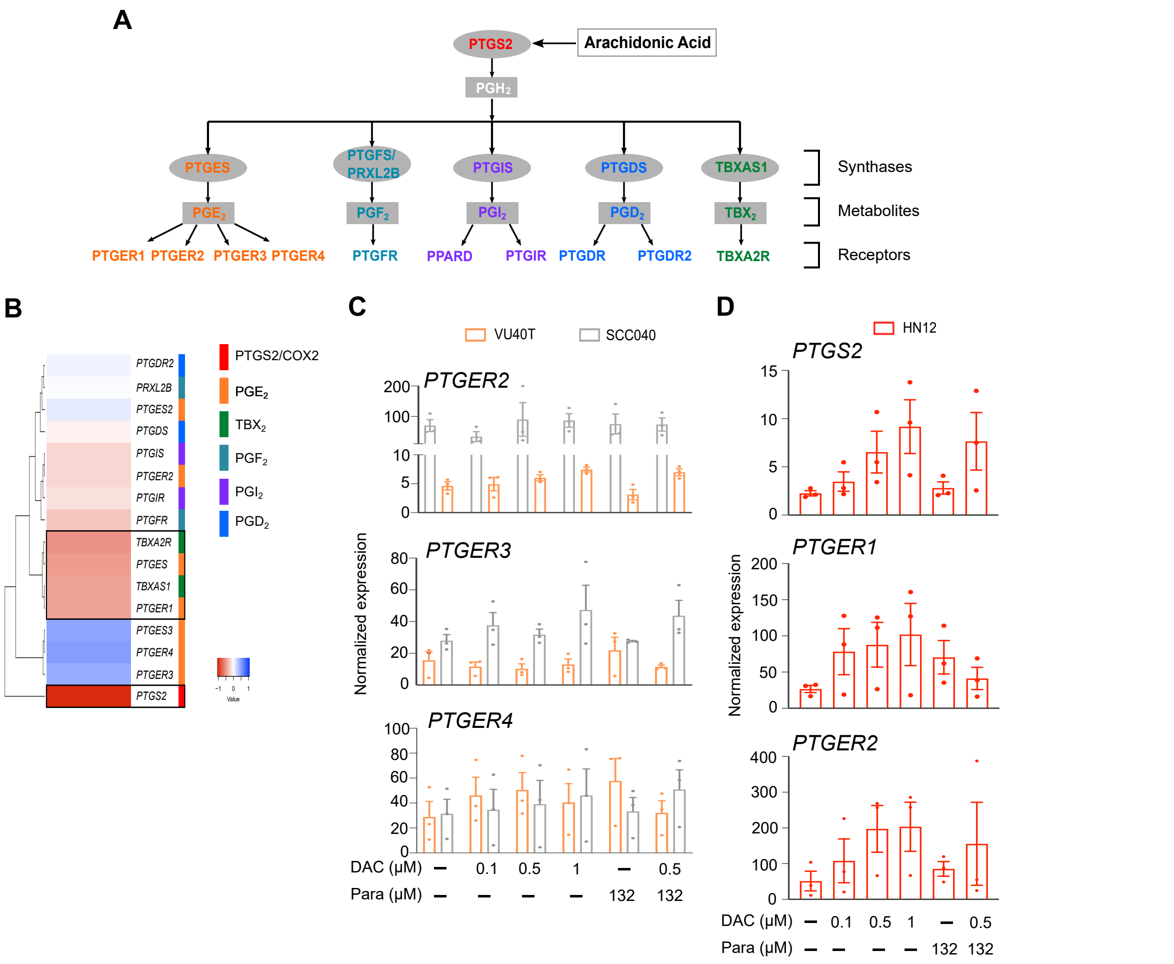

Figure S2. The effects of DAC treatment on COX-2 pathway (related to Fig. 3).

A. Schematic representation of the cyclooxygenase pathway. COX enzymes convert arachidonic acid (AA) into prostaglandin H_2 which is then converted to prostanoids (prostaglandins PGE_2, PGF_2, PGI_2, PGD_2 and thromboxane TBX_2) by respective synthases. Prostanoids are then recognized by G-protein coupled receptors in both autocrine and paracrine manner.

B. DAC-induced expression fold changes for genes encoding synthases and receptors involved in all cyclooxygenase pathways. Data were obtained through RNA-seq in VU40T cells treated with 500 nM DAC for 96h. Black boxes indicate genes with highest upregulation. Colour-coded association with a specific pathway is shown to the right.

C. qRT-PCR for *PTGER2-4* genes in VU40T and SCC040 cells treated for 96h with DAC and/or paracetamol as indicated. Results are normalized to cDNA concentration. While *PTGR1* expression is increased by DAC treatment in DAC-sensitive VU40T cells (Fig. 3D), none of the receptors are up-regulated in DAC-resistant SCC040 cells.

D. qRT-PCR for *PTGS2*, *PTGER1* and *PTGER2* genes in HN12 cells treated for 96h with DAC and/or paracetamol as indicated. Results are normalized to cDNA concentration. Although not statistically significant for each concentration, the expression pattern is consistently up-regulated across all DAC concentrations used. The statistical analysis performed as in Fig. 3A, D.
