# Supplementary material for: The anti-tumour activity of DNA methylation inhibitor 5-aza-2′-deoxycytidine is enhanced by the common analgesic paracetamol through induction of oxidative stress
Source: Cancer Lett. 2021 Mar 31;501:172–86. doi: 10.1016/j.canlet.2020.12.029 (PMC7845757; doi:10.1016/j.canlet.2020.12.029)
Supplement: Multimedia component 3 [file mmc3.pdf]

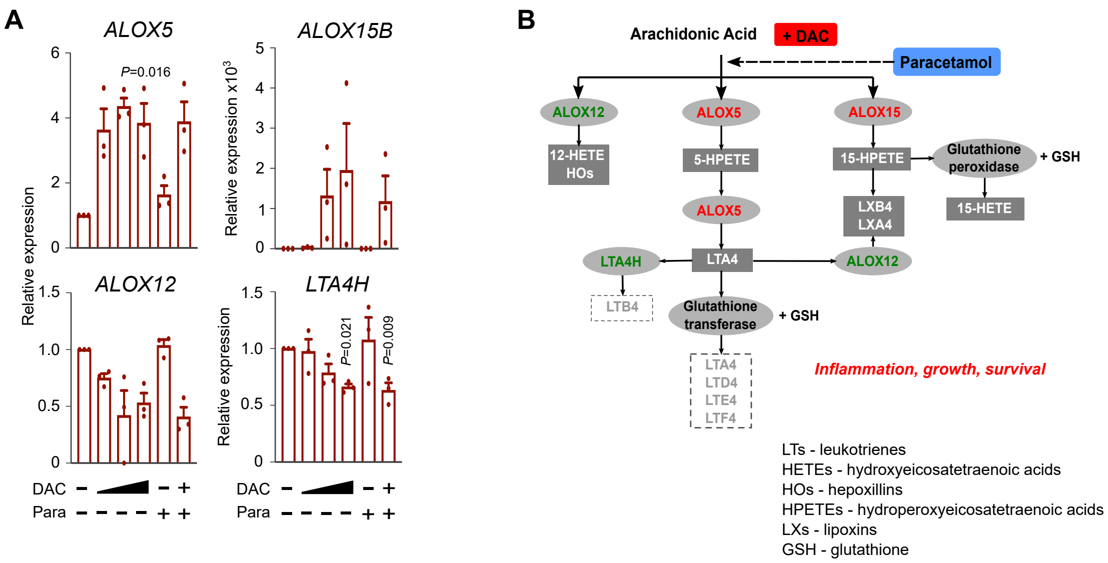

Figure S3. The effects of DAC and paracetamol treatments on LOX pathway.

The possibility that blocking COX-2 pathway with paracetamol could shunt the arachidonic acid towards the LOX pathway was investigated. If confirmed this could also lead to increased cancer cell survival.

A. qRT-PCR for LOX pathway enzymes: *ALOX5*, *ALOX15B*, *ALOX12* and *LTA4H* in VU40T cells treated for 96h as indicated. Data are shown as relative to vehicle control (Ctrl=1). DAC treatment altered gene expression of enzymes involved in the LOX pathway by both up- (*ALOX5* and *ALOX15*) and down- (*ALOX12* and *LTA4H*) regulating them.

B. Schematic of LOX pathway with confirmed DAC effects on gene expression (up-regulation in red, down-regulation in green). However, the secretion of cysteinyl leukotrienes and leukotriene B_4 (shown in dotted boxes) remained below levels detectable by ELISA in all experimental conditions. Therefore there is no indication for LOX pathway compensation following COX-2 inhibition by paracetamol.

In A: n=3, for each cell line a matched One-Way ANOVA with Dunnett's correction to compare all treatments to Ctrl. Values displayed as means +/-SEM. Only significant p-values are shown.
