# Supplementary material for: The anti-tumour activity of DNA methylation inhibitor 5-aza-2′-deoxycytidine is enhanced by the common analgesic paracetamol through induction of oxidative stress
Source: Cancer Lett. 2021 Mar 31;501:172–86. doi: 10.1016/j.canlet.2020.12.029 (PMC7845757; doi:10.1016/j.canlet.2020.12.029)
Supplement: Multimedia component 4 [file mmc4.pdf]

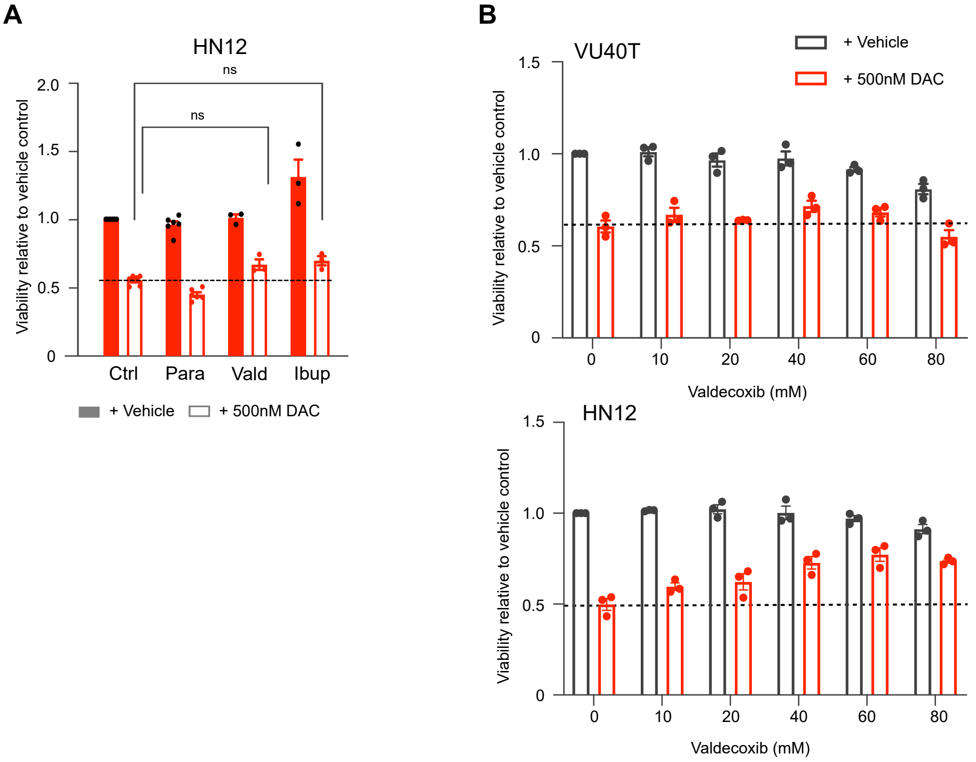

Figure S4. The mechanisms of DAC-paracetamol synergy are specific to paracetamol (related to Fig. 4)**.**
A. Similarly to what was oserved in VU40T cells (Fig. 4A), neither valdecoxib nor ibuprofen sensitizes HN12 cells to DAC treatment. Cell viability in HN12 cells was assessed after 96h treatment with 132.3 µM paracetamol, 10 µM valdecoxib or 193.9 µM ibuprofen +/- 500 nM DAC. The results are shown relative to vehicle control. Dotted line shows the effect of DAC alone. Mean ±SEM; n=3. Statistical analysis as in Fig. 4A.
B. The lack of sensitizing effect of valdecoxib can be observed across a wide range of concentrations. VU40T and HN12 cell viability was assessed after 96h of treatment with indicated concentrations of valdecoxib with or without 500 nM DAC. The results are shown as in (A). Mean ±SEM; n=3.
