# Supplementary material for: The anti-tumour activity of DNA methylation inhibitor 5-aza-2′-deoxycytidine is enhanced by the common analgesic paracetamol through induction of oxidative stress
Source: Cancer Lett. 2021 Mar 31;501:172–86. doi: 10.1016/j.canlet.2020.12.029 (PMC7845757; doi:10.1016/j.canlet.2020.12.029)
Supplement: Multimedia component 5 [file mmc5.pdf]

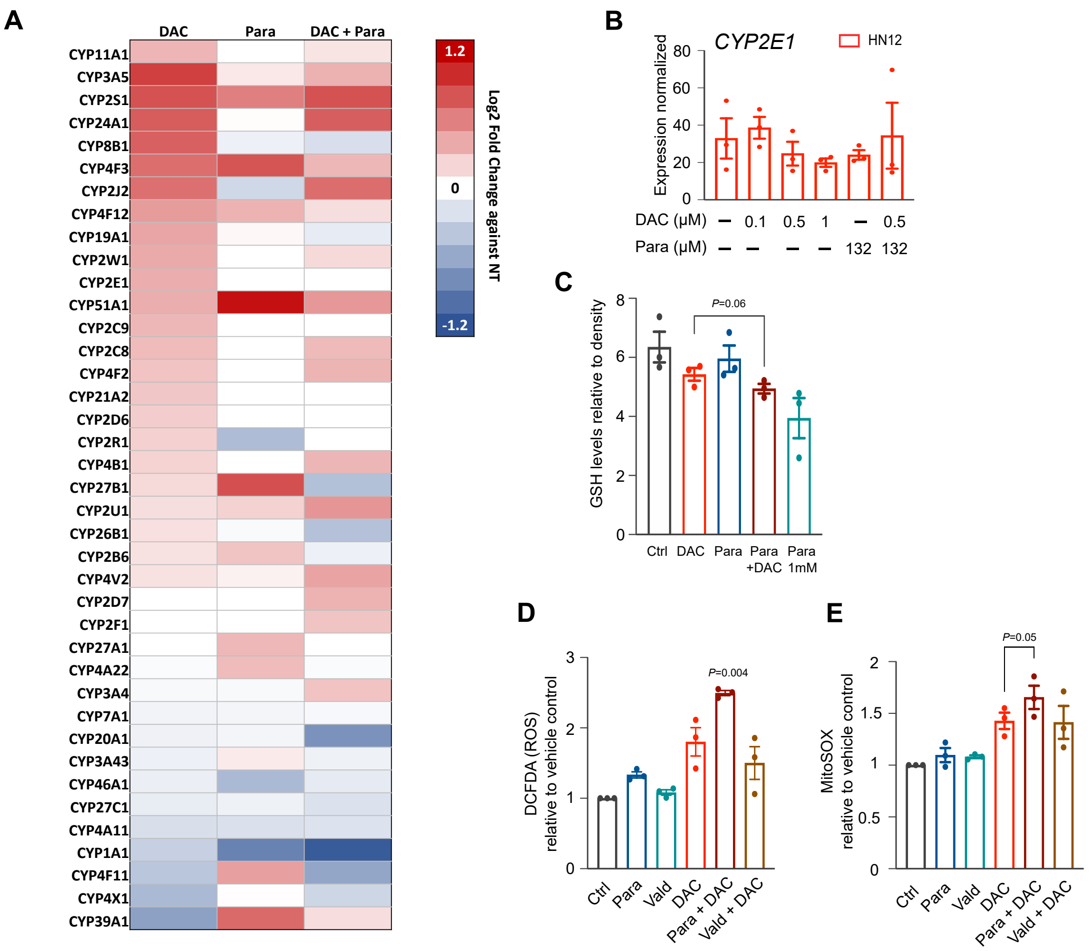

Figure S5. DAC-paracetamol co-treatment leads to GSH depletion and accumulation of reactive oxygen species (related to Fig. 4)**.**

A. Gene expression changes (RNA-seq data) in VU40T cells of CYP enzymes shown as heatmap of $\log_2$ fold change values after indicated treatments (DAC, paracetamol, DAC+paracetamol) against the untreated control. DAC upregulates many genes involved in CYP450 metabolism, some of which could potentially metabolise paracetamol to its toxic product.

B. Although *CYP2E1* is not up-regulated upon DAC in HN12 cells, the involvement of other *CYP* genes is possible, similarly to what is shown in (A). *CYP2E1* qRT-PCR data following 96h treatment of HN12 cells with 500 nM DAC, 132.3 µM paracetamol or both. The results are shown relative to cDNA concentration. n=3, mean +/-SEM.

C-E. Similarly to what was observed in VU40T, in HN12 cells the treatment with DAC+paracetamol significantly affects GSH, ROS and mitochondrial superoxide levels compared to DAC alone.

C. Following DAC+paracetamol treatment, HN12 GSH levels are reduced comparably to 1 mM paracetamol and significantly more than in DAC alone. The cells were treated as above and the results were normalized to cell density. One-Way ANOVA with Dunnett's correction was used to compare all groups to control; a separate two-tailed *t* test was used to compare DAC to DAC+paracetamol.

D-E. The levels of intracellular ROS (**D**) and mitochondrial superoxide (**E**) are increased in HN12 cells upon DAC+paracetamol treatment compared to DAC alone, the effect not observed for DAC+valdecoxib. The cells were treated for 72h as indicated. The results are shown as geometric mean of DCFDA staining (**D**) or MitoSOX Red staining (**E**) normalized to vehicle control. Values are displayed as means +/-SEM, n=3. A matched One-Way ANOVA with Dunnett's correction was used to compare all groups to control; a separate ANOVA was used to compare DAC to DAC+paracetamol and DAC+valdecoxib (Vald).
