# Supplementary material for: The anti-tumour activity of DNA methylation inhibitor 5-aza-2′-deoxycytidine is enhanced by the common analgesic paracetamol through induction of oxidative stress
Source: Cancer Lett. 2021 Mar 31;501:172–86. doi: 10.1016/j.canlet.2020.12.029 (PMC7845757; doi:10.1016/j.canlet.2020.12.029)
Supplement: Multimedia component 6 [file mmc6.pdf]

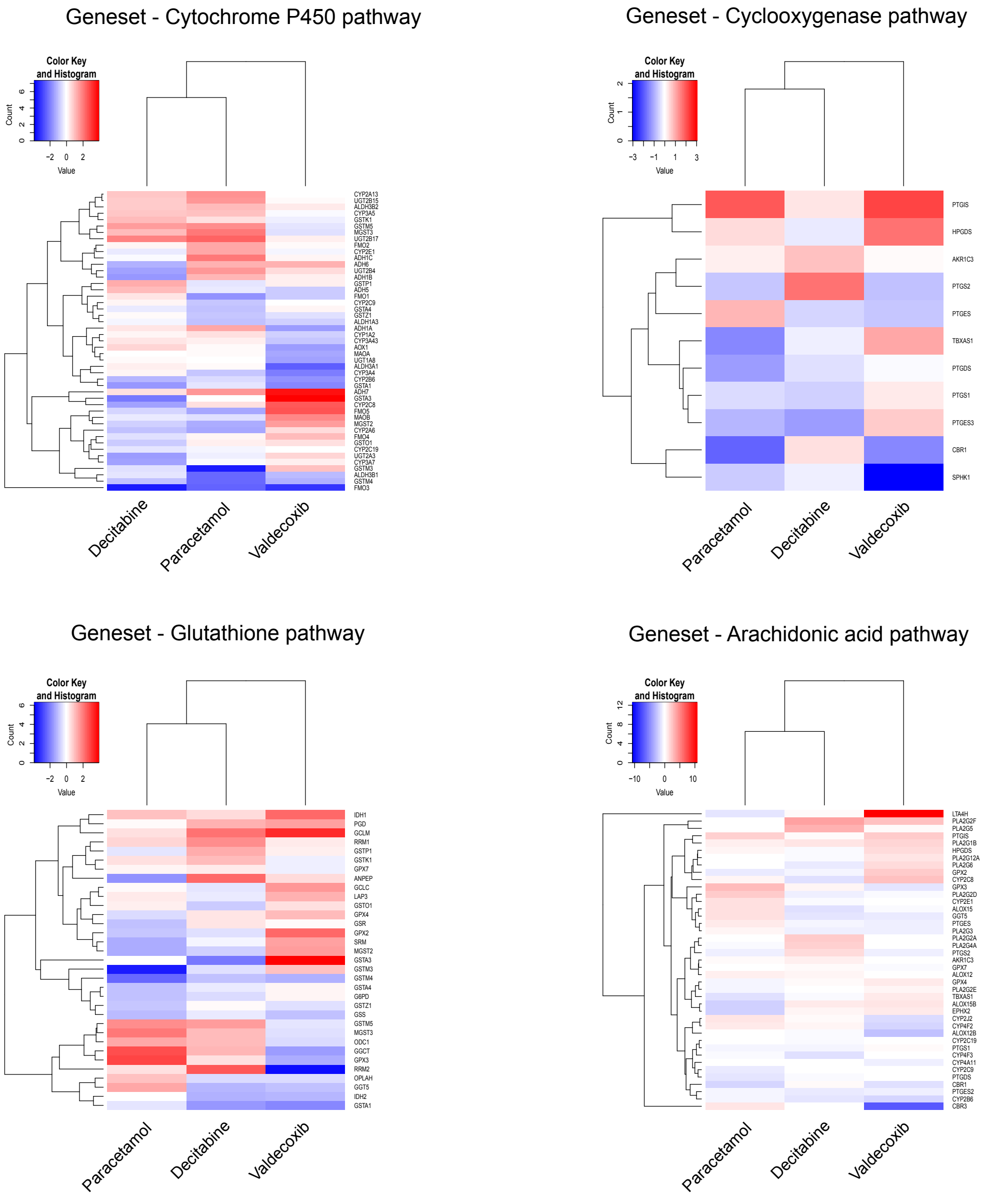

Figure S6. DAC and paracetamol gene expression signatures share similar pathway enrichment. Drug perturbation signatures of Decitabine, paracetamol and valdecoxib, plotted for subsets of genes representing key pathways of interest that were identified from the DSEA analysis. Genes pertaining to each pathway were subsequently obtained from the corresponding genesets identified in MSigDB collection. Clustering of the drug perturbation profiles across these pathways indicates that Decitabine and paracetamol share similar drug perturbation profiles, compared to valdecoxib.
