# Supplementary material for: The anti-tumour activity of DNA methylation inhibitor 5-aza-2′-deoxycytidine is enhanced by the common analgesic paracetamol through induction of oxidative stress
Source: Cancer Lett. 2021 Mar 31;501:172–86. doi: 10.1016/j.canlet.2020.12.029 (PMC7845757; doi:10.1016/j.canlet.2020.12.029)
Supplement: Multimedia component 7 [file mmc7.pdf]

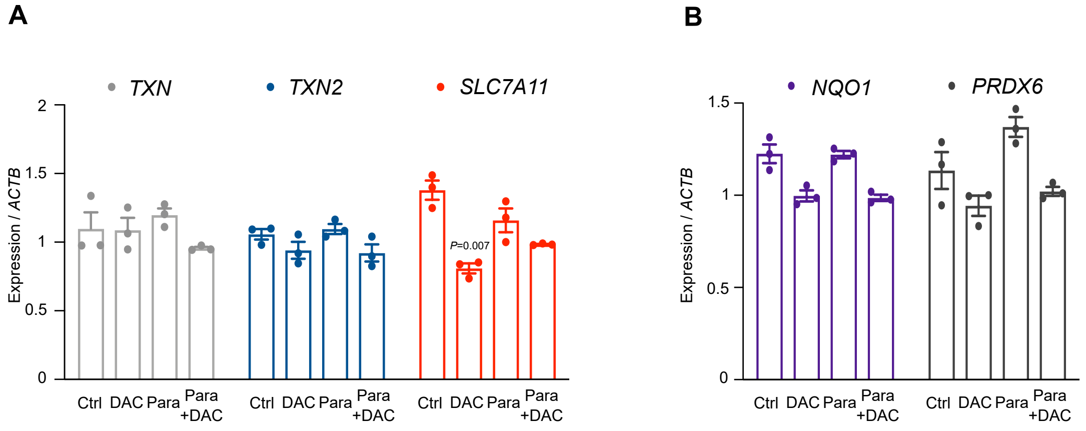

Figure S7. Down-regulation of thioredoxin and anti-oxidant response by DAC treatment in FaDu cells (related to Fig. 5).

RNA-seq data from DAC and DAC+paracetamol treated VU40T cells showed a pattern of down-regulation of genes involved in thioredoxin and anti-oxidant response (Fig. 4J-K). Similar results are observed in qRT-PCR data for FaDu cells.

A-B. qRT-PCR data for genes involved in thioredoxin response (**A:** *TXN*, *TXN2* and *SLC7A11*) and in anti-oxidant response (**B:** *NQO1* and *PRDX6*) also show a moderate but consistent decrease in their expression in FaDu cells. The 96h treatment was performed with 500 nM DAC, 132.3 µM paracetamol or both. The results are shown relative to *ACTB*. For each gene a matched One-Way ANOVA with Dunnett's was used to compare treatments with Ctrl; n=3, mean +/-SEM.
