# Supplementary material for: The anti-tumour activity of DNA methylation inhibitor 5-aza-2′-deoxycytidine is enhanced by the common analgesic paracetamol through induction of oxidative stress
Source: Cancer Lett. 2021 Mar 31;501:172–86. doi: 10.1016/j.canlet.2020.12.029 (PMC7845757; doi:10.1016/j.canlet.2020.12.029)
Supplement: Multimedia component 8 [file mmc8.pdf]

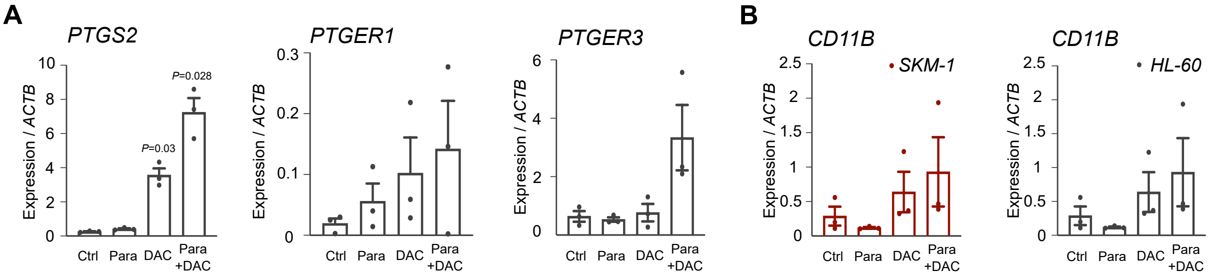

Figure S8. DAC-induced gene expression changes in HL-60 AML cells (related to Fig. 6)**.**

A. DAC treatment significantly up-regulates expression of *PTGS2* gene. qRT-PCR for *PTGS2*, *PTGER1* and *PTGER3* following 72h treatment of HL-60 cells as indicated. Results are normalized to *ACTB* and shown as means +/-SEM, n=3. Statistical analysis as of Fig. 6E.

B. DAC treatment also increases the expression of myeloid differentiation marker *CD11B* (*ITGAM*) in SKM-1 and HL-60 cells. qRT-PCR data shown as in (A).
