# Supplementary material for: The anti-tumour activity of DNA methylation inhibitor 5-aza-2′-deoxycytidine is enhanced by the common analgesic paracetamol through induction of oxidative stress
Source: Cancer Lett. 2021 Mar 31;501:172–86. doi: 10.1016/j.canlet.2020.12.029 (PMC7845757; doi:10.1016/j.canlet.2020.12.029)
Supplement: Multimedia component 9 [file mmc9.docx]

Table S1. qRT-PCR primers’ sequences.

| Gene | Forward (5’-3’) | Reverse (5’-3’) |
| --- | --- | --- |
| *DNMT1* | GAGCCACAGATGCTGACAAA | GACACAGGTGACCGTGCTTA |
| *DNMT3A* | AAGGAGGAGCGCCAAGAG | GGATGGGGACTTGGAGATCA |
| *DNMT3B* | GGGAGGTGTCCAGTCTGCTA | GGCTTTCTGAACGAGTCCTG |
| *TP63* | GTTTCGACGTGTCCTTCCAG | TCTGGATGGGGCATGTCTTT |
| *KRT5* | TGAGGTCAAGGCCCAGTATG | ATCTCATGCTTGGTGTTGCG |
| *IVL* | AACACAAAGGGATCAGCAGC | GCTCCAACAGTTGCTCTTTCT |
| *PTGS2 (COX-2)* | TCATCATCAGCGCCCTCAA | GCTCGTTCACAGCCTTCATG |
| *PTGER1 (EP-1)* | GCCAGCTTGTCGGTATCATG | CTGCAGGGAGGTAGAGCTC |
| *PTGER2 (EP-2)* | AAGCTGTGGTCAAGGCTACA | GCCAAGTACCATGCTCACTG |
| *PTGER3 (EP-3)* | GGATCATGTGCGTGCTGTC | TGTGTCTTGCAGTGCTCAAC |
| *PTGER4 (EP-4)* | TGCTCATCTGCTCCATCCC | ATTCGGATGGCCTGCAAATC |
| *ALOX5* | ACATCTACCTCAGCCTCGTG | AGTTCCTCGTCCACAGTCAC |
| *ALOX15B* | AAATCAAGGGGTTGCTGGAC | AACTGGGAGGCGAAGAAGG |
| *ALOX12* | CTTGCTGAACACTCACCTGG | ATGGTGTAGCGGATATGGGG |
| *LTA4H* | GACTTCTGGGAAGGAACACC | TGCCACCAGTTCTTTAGGGA |
| *CYP2E1* | CGGAACTATGGGATGGGGAA | CGGAAGAGGATGTCGGCTAT |
| *ITGAM (CD11b)* | TGTTTCACGGAACCTCAGGA | ATCCATTGTGAGGTCCTGGC |
| *ACTB* | AAAGACCTGTACGCCAACAC | GTCATACTCCTGCTTGCTGAT |
| *TXN* | AGACTCCAGCAGCCAAGATG | ATCATTTTGCAAGGCCCACA |
| *TXN2* | GGGCCGAGGTTAGAGAAGAT | ACCACGTCCCCATTCTTCAT |
| *SLC7A11* | CTGGCATTTGGACGCTACAT | AAGAAAATCTGGATCCGGGC |
| *NQO1* | AGAAAGGATGGGAGGTGGTG | GAAAGTTCGCAGGGTCCTTC |
| *PRDX6* | GTGTTGAGGACCATCTTGCC | GGCATGCCCTTTTCATCCTT |

Table S2. Dose Reduction Index (DRI) for VU40T, combined treatment.

| Fa  (%) | VU40T | | | |
| --- | --- | --- | --- | --- |
|  | **Dose** | | **DRI**^1^ | |
|  | **DAC** | **Para** | **DAC** | **Para** |
| 10 | 0.01 | 105 | 0.30 | 10.18 |
| 25 | 0.16 | 251 | 1.22 | 7.11 |
| 50 | 2.26 | 595 | **4.99** | **4.97** |
| 75 | 31 | 1414 | 20.35 | 3.47 |
| 97 | 9300 | 9200 | 426 | 1.59 |

^1^DRI values indicate how many times less each drug can be used when in combination.

Table S3. Dose Reduction Index (DRI) for HN12, combined treatment.

| Fa  (%) | HN12 | | | |
| --- | --- | --- | --- | --- |
|  | **Dose** | | **DRI**^1^ | |
|  | **DAC** | **Para** | **DAC** | **Para** |
| 10 | 0.004 | 186 | 0.49 | 80 |
| 25 | 0.06 | 1962 | 1.19 | 147 |
| 50 | 0.85 | 20653 | **2.92** | **269** |
| 75 | 11.92 | 217412 | 7.15 | 493 |
| 97 | 3630 | 3.55E+07 | 49.5 | 1827 |

^1^DRI values indicate how many times less each drug can be used when in combination.

Table S4. Differentially expressed (increased and decreased) genes in VU40T cells following DAC, paracetamol and DAC+paracetamol treatments. See the Table_S4.xlsx file.

Table S5. Full list of GO terms from REVIGO for differentially expressed groups (DAC, paracetamol and DAC+paracetamol treatments). See the Table_S5.xlsx file.

Table S6. cBioPortal RNA-seq data (for cancers with provisional TCGA data): frequency of expression alterations (%) in genes from COX-2-PGE_2_ pathway and correlation to survival (Logrank test p-value).

| Cancer type | n | PTGS2 | PTGES | PTGES2 | PTGES3 | PTGER1 | PTGER2 | PTGER3 | PTGER4 | OS | DFS |
| --- | --- | --- | --- | --- | --- | --- | --- | --- | --- | --- | --- |
| Adrenocortical ca. | 79 | 1.3 | 4 | 5 | 19 | 9 | 1.3 | 5 | 6 | 0.181 | 0.130 |
| Cholangiocarcinoma | 36 | 6 | 6 | 6 | 2.8 | 8 | 2.8 | 2.8 | 2.8 | 0.322 |  |
| Bladder urothelial ca. | 408 | 4 | 3 | 2.5 | 8 | 5 | 3 | 4 | 8 | 0.0702 |  |
| Colorectal adenoca. | 379 | 3 | 6 | 1.6 | 7 | 2.4 | 4 | 4 | 4 | 0.0375 |  |
| Breast invasive ca. | 1093 | 1.7 | 4 | 4 | 8 | 1.6 | 2.6 | 2.8 | 3 | 0.259 |  |
| Glioblastoma mult. | 160 | 3 | 6 | 6 | 8 | 6 | 4 | 6 | 3 |  |  |
| Cervical SCC | 304 | 2.6 | 6 | 4 | 9 | 5 | 4 | 8 | 6 | 0.17* |  |
| Esophageal carcinoma | 184 | 7 | 2.7 | 8 | 8 | 5 | 6 | 1.6 | 9 |  |  |
| Stomach adenoca. | 415 | 5 | 4 | 9 | 9 | 4 | 0.7 | 4 | 7 |  | 0.09* |
| Uveal melanoma | 80 | 5 | 2.5 | 6 | 6** | 5 | 5 | 5 | 5 |  |  |
| HNSCC | 520 | 2.3 | 4 | 7 | 7 | 2.5 | 4 | 3 | 6 | 0.090 | 5.693e-3 |
| Kidney renal clear cell ca | 533 | 1.3 | 2.3 | 2.4 | 5 | 0.4 | 4 | 7 | 5 | 0.179 | 0.144 |
| Kidney renal papillary cell ca. | 290 | 2.4 | 4 | 4 | 9 | 3 | 4 | 2.4 | 4 | 0.034 | 0.307 |
| Liver hepatocellular ca. | 371 | 0.8 | 6 | 7 | 8 | 6 | 3 | 1.3 | 4 | 0.283 |  |
| Lung adenocarcinoma | 515 | 4 | 4 | 3 | 11 | 2.1 | 5 | 3 | 4 | 0.039 |  |
| Lung SCC | 501 | 4 | 6 | 3 | 5 | 2.4 | 1.2 | 1.4 | 7 |  | 0.202 |
| AML | 173 | 4 | 5 | 4 | 2.9 | 2.3 | 3 | 2.9 | 2.3 |  | n/a |
| Ovarian serous cystadenoca. | 307 | 0.7 | 2.6 | 7 | 4 | 2.3 | 2 | 5 | 5 | 2.305e-3 | 0.286 |
| Pancreatic adenoca. | 178 | 4 | 4 | 1.7 | 10 | 3 | 4 | 6 | 4 | 0.089 | 0.080 |
| Mesothelioma | 87 | 3 | 1.1 | 1.1 | 10 | 8 | 1.1 | 3 | 5 | n/a | n/a |
| Prostate adenoca. | 497 | 2.8 | 5 | 6 | 5 | 4 | 4 | 5 | 3 |  | 0.262 |
| Skin cutaneous melanoma | 469 | 8 | 1.7 | 3 | 9 | 4 | 4 | 2.1 | 3 | 0.118 | 0.236 |
| Sarcoma | 259 | 5 | 10 | 6 | 8 | 5 | 8 | 4 | 5 |  |  |
| Testicular germ cell ca. | 150 | 2 | 7 | 7 | 9 | 5 | 6 | 3 | 3 |  | 0.072 |
| Thymoma | 120 | 5 | 3 | 3 | 8** | 4 | 4 | 3 | 4 | 0.017 | 0.290 |
| Thyroid cancer | 501 | 3 | 2.4 | 4 | 6 | 3 | 3 | 3 | 5 |  |  |
| Uterine corpus endothelial carc. | 177 | 1.7 | 2.8 | 5 | 6 | 4 | 7 | 3 | 6 |  |  |
| Overall % |  | **3.4** | **4.3** | **4.7** | **7.7** | **4.1** | **3.7** | **3.7** | **4.8** |  |  |

OS, Overall Survival, DFS, Disease/Progression Free Survival. Only p-values < 0.35 are shown. OS and DFS values describe negative impact on survival, unless marked by * (correlation with better survival). Expression alterations are predominantly observed as overexpression unless marked by ** (where downregulation is observed). SCC, squamous cell carcinoma.

Table S7. cBioPortal RNA-seq data (for cancers with provisional TCGA data): frequency of expression alterations (%) in genes involved in glutathione synthesis and correlation to survival (Logrank test p-value).

| Cancer type | n | GCLC | GCLM | GSS | GGCT | OPLAH | GSR | OS | DFS |
| --- | --- | --- | --- | --- | --- | --- | --- | --- | --- |
| Adrenocortical ca. | 79 | 5 | 1.3 | 6 | 16 | 6 | 4 | 0.093 |  |
| Cholangiocarcinoma | 36 | 8 | 2.8 | 2.8 | 11 | 8 | 6 |  | 0.296 |
| Bladder urothelial ca. | 408 | 4 | 4 | 18 | 16 | 11 | 2.9 | 6.327e-3 | 0.231 |
| Colorectal adenoca. | 379 | 8 | 4 | 39 | 20 | 7 | 16** |  |  |
| Breast invasive ca. | 1093 | 5 | 7 | 10 | 8 | 15 | 4 | 0.017 | 0.339 |
| Glioblastoma mult. | 160 | 4 | 4 | 14 | 31 | 3 | 8 | 0.175 | 0.087 |
| Cervical SCC | 304 | 3 | 3 | 12 | 8 | 8 | 4 |  |  |
| Esophageal carcinoma | 184 | 7 | 4 | 13 | 16 | 11 | 8 |  |  |
| Stomach adenoca. | 415 | 7 | 6 | 14 | 13 | 15 | 9 | 0.04* | 0.163* |
| Uveal melanoma | 80 | 13 | 1.3 | 8 | 6 | 35 | 6 |  |  |
| HNSCC | 520 | 6 | 6 | 10 | 8 | 12 | 6 | 0.019 | 2.763e-3 |
| Kidney renal clear cell ca | 533 | 5 | 4 | 6 | 8 | 5 | 5 | 0.185 | 0.070 |
| Kidney renal papillary cell ca. | 290 | 4 | 4 | 16 | 7 | 8 | 3 | 0.051 | 0.041 |
| Liver hepatocellular ca. | 371 | 6 | 3 | 5 | 7 | 20 | 2.7 |  |  |
| Lung adenocarcinoma | 515 | 6 | 2.9 | 8 | 5 | 14 | 5 | 0.035 |  |
| Lung SCC | 501 | 7 | 7 | 10 | 10 | 12 | 5 |  | 0.211* |
| AML | 173 | 5 | 6 | 4 | 3 | 6 | 7 |  |  |
| Ovarian serous cystadenoca. | 307 | 4 | 4 | 13 | 4 | 35 | 3 |  | 0.337* |
| Pancreatic adenoca. | 178 | 4 | 2.2 | 8 | 7 | 7 | 5 | 0.15* |  |
| Mesothelioma | 87 | 7 | 3 | 6 | 3 | 9 | 6 | n/a | n/a |
| Prostate adenoca. | 497 | 6 | 4 | 4 | 8 | 11 | 6** | 0.13* | 7.141e-3 |
| Skin cutaneous melanoma | 469 | 13 | 5 | 11 | 18 | 8 | 8 | 2.585e-3 | 0.184 |
| Sarcoma | 259 | 2.7 | 4 | 9 | 12 | 3 | 5 | 0.011 | 0.041 |
| Testicular germ cell ca. | 150 | 7 | 13 | 2.7 | 26 | 4 | 9 ** | 0.05 |  |
| Thymoma | 120 | 6 | 3 | 7 | 6 | 4 | 5 | 1.111e-4 |  |
| Thyroid cancer | 501 | 2.4 | 1.8 | 6 | 4 | 1.4 | 1.6 |  |  |
| Uterine corpus endothelial carc. | 177 | 5 | 4 | 6 | 6 | 12 | 3 |  |  |
| Overall % |  | **5.9** | **4.2** | **9.9** | **10.6** | **10.8** | **5.7** |  |  |

OS, Overall Survival, DFS, Disease/Progression Free Survival. Only p-values < 0.35 are shown. OS and DFS values describe negative impact on survival, unless marked by * (correlation with better survival). Expression alterations are predominantly observed as overexpression unless marked by ** (where downregulation is observed). SCC, squamous cell carcinoma.
