# Supplementary material for: The anti-tumour activity of DNA methylation inhibitor 5-aza-2′-deoxycytidine is enhanced by the common analgesic paracetamol through induction of oxidative stress
Source: Cancer Lett. 2021 Mar 31;501:172–86. doi: 10.1016/j.canlet.2020.12.029 (PMC7845757; doi:10.1016/j.canlet.2020.12.029)
Supplement: Multimedia component 10 [file mmc10.docx]

# Supplementary Methods

Detailed information is provided for the following methods and procedures: DNA dot blotting, Western blot analysis, immunofluorescence analysis, apoptosis assay, ROS and mitosox assessment, qRT-PCR, RNA-seq data analysis, mouse xenograft study, AML cells’ growth rate assessment, TCGA data analysis and drug perturbation signatures.

#### Dot blotting of DNA

DNA was extracted using the DNeasy Blood and Tissue Kit (Qiagen) as described by the manufacturer. Dot blotting was performed as described in Current Protocols in Molecular Biology using a titration of DNA (Brown, 2001). Dried membranes (Nytran Supercharge Positively Charged Nylon Membrane (Fisher Scientific)) were blocked in 5% low-fat milk in PBS for 1h and incubated with primary antibody (anti-5mC, Cell Signalling (28692S), 1:750 dilution) in 1% bovine serum albumin (BSA) at 4°C overnight. Membranes were incubated with secondary antibody (Santa Cruz, Cat# sc-2004, 1:5000 dilution) for 2h at 4°C. Membranes were developed using 1:1 Amersham ECL western blotting reagent (GE Healthcare) to SuperSignal West Femto Chemiluminescent Substrate (Thermo Fisher Scientific) and detected by autoradiography. Blots were stained for total DNA by 30min incubation in 0.04% methylene blue (Sigma-Aldrich). ImageJ software (Schindelin *et al*, 2012) was used to quantify the intensity of the 1 μg and 0.5 μg dots and each 5-methylcytosine dot was normalised against its methylene blue counterpart. For each biological replicate an average was taken from the normalised 1 μg and 0.5 μg dots and the technical replicates for use in further analysis.

#### Western Blot analysis

Cell pellets were incubated with RIPA buffer (150mM sodium chloride, 0.5% sodium deoxycholate, 0.1% sodium dodecyl sulphate, 50mM Tris-HCl pH8) supplemented with protease inhibitors (Roche) for 45min then centrifuged at 17,000g for 10min and the supernatant was retained. Proteins were denatured in Laemmli buffer (4% SDS, 20% glycerol, 10% 2-mercaptoethanol, 0.004% bromophenol blue and 0.125 M Tris HCl), heated to 95°C for 5min and separated on a 10% SDS-PAGE gel before transferring onto a nitrocellulose membrane (Amersham Hybond ECL, GE Healthcare) using BioRad transfer apparatus. The protein transfer was confirmed with Ponceau S staining (SigmaAldrich).

Membranes were blocked in 20% milk in PBST for 1h following primary antibody incubation (COX-2: Abcam, Cat# ab15191, 1:1000 dilution, 1h at room temperature in 1% BSA; CYP2E1: Abcam, Cat# ab28146, 1:2500 dilution, overnight 4°C in 5% milk; lamin A/C: Santa Cruz, Cat# sc-20681, 1:10,000, 1h at room temperature in 5% milk). Membranes were incubated with secondary antibody (anti-rabbit-IgG-HRP Santa Cruz, Cat# sc-2004, 1:5000 dilution) for 2h and developed as described for DNA dot blotting. ImageJ software was used to quantify the intensity of each band and bands of interest were normalised against the corresponding lamin A/C bands.

#### Immunofluorescence analysis

Immunofluorescence analysis was undertaken as described in (Roulois *et al*, 2015). For γH2AX staining, cells were first incubated with ice-cold pre-extraction buffer (10 mM PIPES pH 6.8, 300 mM sucrose, 20 mM NaCl, 3 mM MgCl_2_ 0.5% Triton X-100) for 7min. For both γH2AX and Ki67 staining the cells were fixed in methanol for 15min, followed by 1min incubation with ice-cold acetate. Samples were blocked with 1% BSA in PBS for 1h at 4^o^C following incubation with the primary antibodies overnight (Ki67 (Abcam, Cat# ab15580) 1:1000; γH2AX (phospho S139) (Abcam, Cat# ab2893) 1:1000). Coverslips were incubated with secondary antibody (Jackson ImmunoResearch, Donkey anti-rabbit Alexa Fluor 488 cat# 711-545-152, 1:250) for 1h at room temperature, washed with PBS before drying and mounting using ProLong Gold Antifade Mountant with DAPI (Fisher Scientific). Cells were stored in the dark for 3 days prior to imaging on a Zeiss 780 Zen confocal microscope. For Ki67 staining, ImageJ was used to count percentage of positive cells in triplicate images from each biological replicate. For γH2AX staining, the number of γH2AX per nuclei was counted manually across all focal planes. 100 nuclei were analysed in each biological replicate.

#### Apoptosis assay

Following flow cytometry for cells stained with annexin V and propidium iodide (PI), the cells were gated into four populations and counted: healthy cells which were positive for neither PI nor annexin V; necrotic cells stained only with PI; early apoptotic cells stained only with annexin V and late apoptotic cells stained with both PI and annexin V. The proportion of cells in each quadrant after 48h, 72h, 96h of treatment was counted and compared to the vehicle control.

**Assessment of reactive oxygen species (ROS) and mitochondrial superoxide (mitosox)**

MitoSOX Red (Molecular Probes, Cat# M36008, ThermoFisher Scientific) was used to assess mitosox. PBS-washed cells were resuspended in 200 µl PBS (37°C) containing 5 µM MitoSOX Red, incubated at 37°C for 10 min and then analysed by flow cytometry (emission wavelength 580 nm). Reactive oxygen species (ROS) were measured using carboxy-H_2_DCFDA (5-(and-6)-carboxy-2’,7’-dichlorodihydrofluorescein diacetate, Cat# C369, Invitrogen). 10 µM carboxy-H_2_DCFDA was added to 0.5 ml cell suspension for 45 min, cells were washed with PBS and resuspended in 200 µl PBS (37°C). Resuspended cells were analysed by flow cytometry (emission wavelength 517-527 nm) with BD FACSCalibur and BD Cell Quest software. The geometric mean was calculated for each sample and experimental condition.

#### Real-Time quantitative PCR (qRT-PCR)

The primers used in qRT-PCR are listed in Supplementary Table S1. Relative RNA values were normalised against the concentration of the purified cDNA as measured by Qubit dsDNA High Sensitivity Assay kit (Thermo Fisher Scientific). This method of normalization was treated as an additional step to the normalization to *ACTB* expression because the DAC treatments can lead to decrease of RNA yield and changes in housekeeping gene expression. Each sample was examined at least in triplicate. PCR product specificity was confirmed by a melting-curve analysis.

**RNA sequencing**

Quantification of the RNA sequencing data was performed according to the latest recommended pipeline as defined in the DeSeq2 software. A count for each gene was calculated using the reference-free aligner Salmon (Patro *et al*, 2017) and the resulting count table was processed using DeSeq2 (Love *et al*, 2014) to compare treatment groups. There were 1200 upregulated (log2 fold change ≥1) and 937 downregulated (log2 fold change ≤-1) genes, further divided into six groups: i) genes up-regulated by paracetamol (n=151); ii) genes down-regulated by paracetamol (n=127); iii) genes up-regulated by DAC (n=658); iv) genes down-regulated by DAC (n=293); v) genes up-regulated by combined treatment (n=915); and vi) genes down-regulated by combined treatment (n=721). Each group was subjected to gene ontology (GO) analysis using the Gene Ontology Consortium software (Ashburner *et al*, 2000) followed by removal of redundant terms using REVIGO (Supek *et al*, 2011). The top 5 terms with the Log_10_ p-value ≤3.0 from each group were collated; none of the GO terms from the gene set downregulated by paracetamol fulfilled this condition. The Log_10_ p-values were used to generate the heatmap.

**Mouse xenograft study**

To examine the toxicity and anti-tumour efficacy of DAC and paracetamol, we utilised male NOD/SCID/gamma (NSG) mice (Charles River) which were kept in 12 hour light and 12 hour dark cycles in individually ventilated cages. Mice were maintained on mouse feed and water, ad libitum, and were at least 6 weeks old at the start of the treatments.

Toxicity studies with 3 animals per single treatment and combination treatment were used to identify safe doses for use in subsequent efficacy study: 0.4 mg/kg DAC and 100 mg/kg paracetamol. In the toxicity studies the drugs were given 5 days a week for three weeks, first DAC and paracetamol separately, then in combination.

To assess the efficacy, 24 male NSG mice were implanted with 5x10^6^ FaDu HNSCC cells suspended in serum-free medium and injected subcutaneously into the right flank. Tumours were given three days to become established, at which point mice were randomly allocated into four treatment groups, each group containing 6 animals, as follows: (1) 0.4 mg/kg DAC dissolved in PBS via intraperitoneal (IP) injection on a 5 day on, 2 day off regimen from day 4 onwards; (2) 100 mg/kg paracetamol dissolved in PBS given via oral gavage following the same regimen; (3) DAC plus paracetamol as above; (4) control (PBS) given both through the oral gavage and IP injection. Animals were monitored daily for signs of ill health and the tumours were measured. Tumour measurements were taken using callipers and volumes calculated using the formula L x W^2^. Mice were culled once: tumours reached a maximum of 1250 mm^3^; tumours became ulcerated; animals showed signs of ill health; or at completion of the study. One mouse from group 1 had to be excluded on Day 10 due to dosing error. When displaying tumour growth over time, to gain an understanding of efficacy over a longer period of time, data were plotted until at least 4 animals per group remained; when fewer animals remained in a group, no more data were plotted. The tumour growth is also shown as time to reach volume of 200 mm^3^; tumours smaller than 200 mm^3^ at the end of the study (day 25) were counted as ‘Day 25’.

Tumours were excised and snap frozen for subsequent analysis. Tumour tissue was available from 6 animals in control and paracetamol-treated groups, 4 animals from DAC-treated and 5 animals from DAC+paracetamol treated groups. Approximately 10-20mg tissue was pulverized and total RNA extracted as described before (see: Real-time quantitative PCR).

**Long treatment of AML cells and growth rate assessment**

SKM-1 and HL-60 cells were subjected to a repeated cycle of treatments. The cells were plated in 6-well plates at 0.5x10^6^ cells/ml and 0.375x10^6^ cells/ml for SKM-1 and HL-60, respectively, treated for 72h after which the cells were counted and re-plated without drugs at original concentrations. The withdrawal period lasted 21 days during which the cells were passaged twice a week. The treatment cycle was repeated four times and the cells were counted at each passage using trypan blue (Sigma-Aldrich) staining and haemocytometer cell counting. Growth rate was calculated using the following equation Gr=ln(N(t)/N(0))/t, where Gr = growth rate, N(t)=number of cells at time t, N(0)=number of cells at time 0, and t=time(hours).

**TCGA data**

The following data were retrieved from The Cancer Genome Atlas (TCGA) via cBioPortal for Cancer Genomics (Cerami *et al*, 2012; Gao *et al*, 2013): (i) percentage of samples for each cancer type with mRNA Expression z-score threshold ± 2.0 (RNA Seq V2 RSEM) for all cancers with available TCGA provisional data (Supplementary Tables S6 and S7); (ii) clustered gene expression heatmap for genes in COX-2 pathway in HNSCC tumours (520 patients, Fig. 3F); (iii) Logrank test p-values for overall survival and/or disease/progression-free survival for all cancers with available TCGA provisional data in indicated set of genes (expression z-score threshold ± 2.0), (Supplementary Tables S6 and S7, Fig. 5A-C); (v) Kaplan-Meier curves for HNSCC (520 patients) disease-progression free survival based on gene expression alterations in indicated sets of genes (Fig. 5A-C). The following gene sets have been used: COX-2 pathway (*PTGS2*, *PTGES*, *PTGES2*, *PTGES3*, *PTGER1*, *PTGER2*, *PTGER3*, *PTGER4*); Glutathione synthesis (*GSS*, *GCLC*, *GCLM*, *GGCT*, *OPLAH*, *GSR*); Thioredoxin pathway genes down-regulated by DAC (*TXN2*, *SLC7A11*, *TXN*, *TXNRD1*).

**Drug perturbation signatures**

Drug perturbation signatures were downloaded for the BROAD Connectivity Map dataset (CMAP) using the PharmacoGx package (version 1.14.0) (Smirnov *et al*, 2016) in R. cMap drug perturbation signatures represent transcriptionally profiled, drugs-treated cancer cell lines involving 11,833 genes and 1,309 drugs across 5 cancer cell lines (breast cancer MCF7 and ssMCF7, pancreatic cancer PC3, melanoma SKMEL5 and AML HL-60). Precomputed signatures for CMAP were available for 1288 drugs; the signatures are calculated using a linear regression model adjusted for treatment duration, cell line identity, and batch, as described previously (Smirnov *et al.*, 2016). Heatmaps of drug perturbation signatures (transcriptional profiles) for Decitabine (DAC), paracetamol (Para), and Valdecoxib were plotted using ggplots package (version 3.0.1.1). All analyses have been conducted using the R statistical software (version 3.6.0); listed software dependencies are available on the Comprehensive Repository R Archive Network (CRAN) or Bioconductor (BioC).

**References**

Ashburner M, Ball CA, Blake JA, Botstein D, Butler H, Cherry JM, Davis AP, Dolinski K, Dwight SS, Eppig JT *et al* (2000) Gene ontology: tool for the unification of biology. The Gene Ontology Consortium. *Nat Genet* 25: 25-29

Brown T (2001) Dot and slot blotting of DNA. *Curr Protoc Mol Biol* Chapter 2: Unit2 9B

Cerami E, Gao J, Dogrusoz U, Gross BE, Sumer SO, Aksoy BA, Jacobsen A, Byrne CJ, Heuer ML, Larsson E *et al* (2012) The cBio cancer genomics portal: an open platform for exploring multidimensional cancer genomics data. *Cancer Discov* 2: 401-404

Gao J, Aksoy BA, Dogrusoz U, Dresdner G, Gross B, Sumer SO, Sun Y, Jacobsen A, Sinha R, Larsson E *et al* (2013) Integrative analysis of complex cancer genomics and clinical profiles using the cBioPortal. *Sci Signal* 6: pl1

Love MI, Huber W, Anders S (2014) Moderated estimation of fold change and dispersion for RNA-seq data with DESeq2. *Genome Biol* 15: 550

Patro R, Duggal G, Love MI, Irizarry RA, Kingsford C (2017) Salmon provides fast and bias-aware quantification of transcript expression. *Nat Methods* 14: 417-419

Roulois D, Loo Yau H, Singhania R, Wang Y, Danesh A, Shen SY, Han H, Liang G, Jones PA, Pugh TJ *et al* (2015) DNA-Demethylating Agents Target Colorectal Cancer Cells by Inducing Viral Mimicry by Endogenous Transcripts. *Cell* 162: 961-973

Schindelin J, Arganda-Carreras I, Frise E, Kaynig V, Longair M, Pietzsch T, Preibisch S, Rueden C, Saalfeld S, Schmid B *et al* (2012) Fiji: an open-source platform for biological-image analysis. *Nat Methods* 9: 676-682

Smirnov P, Safikhani Z, El-Hachem N, Wang D, She A, Olsen C, Freeman M, Selby H, Gendoo DM, Grossmann P *et al* (2016) PharmacoGx: an R package for analysis of large pharmacogenomic datasets. *Bioinformatics* 32: 1244-1246

Supek F, Bosnjak M, Skunca N, Smuc T (2011) REVIGO summarizes and visualizes long lists of gene ontology terms. *PLoS One* 6: e21800
